# Once upon a time the cell membranes: 175 years of cell boundary research

**DOI:** 10.1186/s13062-014-0032-7

**Published:** 2014-12-19

**Authors:** Jonathan Lombard

**Affiliations:** National Evolutionary Synthesis Center, 2024 W. Main Street Suite A200, Durham, NC 27705 USA

**Keywords:** Cell membrane discovery, Cell membrane structure, Cell Theory, History of Science, Cell definition, Origins of life, Early evolution, Cenancestor

## Abstract

**Abstract:**

All modern cells are bounded by cell membranes best described by the fluid mosaic model. This statement is so widely accepted by biologists that little attention is generally given to the theoretical importance of cell membranes in describing the cell. This has not always been the case. When the Cell Theory was first formulated in the XIX^th^ century, almost nothing was known about the cell membranes. It was not until well into the XX^th^ century that the existence of the plasma membrane was broadly accepted and, even then, the fluid mosaic model did not prevail until the 1970s. How were the cell boundaries considered between the articulation of the Cell Theory around 1839 and the formulation of the fluid mosaic model that has described the cell membranes since 1972? In this review I will summarize the major historical discoveries and theories that tackled the existence and structure of membranes and I will analyze how these theories impacted the understanding of the cell. Apart from its purely historical relevance, this account can provide a starting point for considering the theoretical significance of membranes to the definition of the cell and could have implications for research on early life.

**Reviewers:**

This article was reviewed by Dr. Étienne Joly, Dr. Eugene V. Koonin and Dr. Armen Mulkidjanian.

## Introduction

Modern descriptions of the cell are intimately related to the notion of cell membranes. The cell membrane is not only the boundary of the unit of life, it is also a specific compartment that harbors many essential cell functions including communication with the environment, transport of molecules and certain metabolic functions. Nowadays, the consensual model to depict the membrane structure and functions is called the “fluid mosaic model” [1].

**Figure 1:**
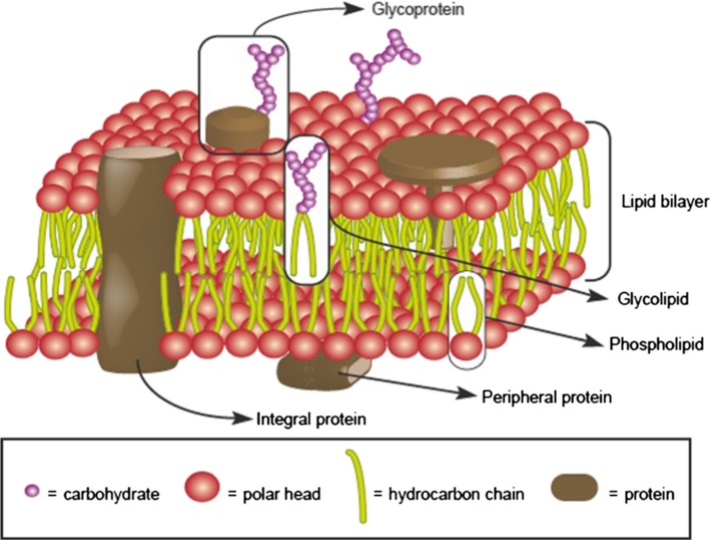
**Fluid mosaic model.** Schematic view of biological membrane structure as currently depicted.

The fluid mosaic hypothesis was formulated by Singer and Nicolson in the early 1970s [1]. According to this model, membranes are made up of lipids, proteins and carbohydrates (Figure 1). The main lipid membrane components are phospholipids. These molecules are amphiphilic, i. e. they have one polar part attracted by water (hydrophilic) and one apolar component repelled by water (hydrophobic). When they are diluted in water, amphiphiles spontaneously adopt the most thermodynamically stable molecular structure, namely the one that maximizes both hydrophilic and hydrophobic interactions [2]. These interactions may be affected by several parameters, such as the chemical nature of the molecules, their size, the salinity and pH of the solution. In biological conditions, cell phospholipids form a bilayer in which hydrophobic tails face each other in the core of the structure whereas the hydrophilic heads interact with the surrounding water (Figure 1). Since proteins are also amphiphilic molecules, the same constraints apply to them. Some proteins (called intrinsic or integral) are embedded in the lipid bilayer matrix where they are able to establish hydrophobic and hydrophilic interactions with their respective lipid counterparts. Other proteins, called extrinsic or peripheral proteins, can also be transiently associated with membrane surfaces through weaker interactions (Figure 1). Finally, carbohydrates can be linked to either proteins or lipids, resulting in glycoproteins or glycolipids.

The “mosaic” term of this model refers to the mixture of lipids and intrinsic proteins in the membrane. These boundaries are also “fluid” because their components can move laterally, allowing both diffusion of components and local specific gatherings. Other lipids, such as cholesterol, act as membrane fluidity regulators. Phospholipid movements are generally restricted to lateral drift, because the cross of the membrane from one side to the other requires the energetically unfavorable transient contact of their hydrophilic head with the hydrophobic membrane core. Thus, the transfer of molecules from one side of the membrane to the other generally involves the activity of some specific integral membrane proteins, called flippases [3]. For the same reasons, integral proteins can diffuse within the lipid matrix but they seldom switch their polarity from one membrane side to the other. As a result, lipid, protein and carbohydrate composition are different between the two monolayers, a characteristic that is referred to as membrane asymmetry.

Membrane functions are extremely diverse. As cell borders, membranes control the molecular exchanges with the environment, resulting in cell pH regulation and osmotic homeostasis. Membranes are “selective barriers”: They concentrate nutrients within the cell, exclude the cellular waste products, keep the ionic gradients and transform them into chemical energy. Since they allow the transduction of many external stimuli into cell signals, they are also major actors in the responses of the cell to their environment. In addition, their composition also turns membranes into the main apolar compartment of the prominently aqueous cell medium, thus concentrating most lipid pigments (e.g. chlorophyll) and hydrophobic proteins. The presence of these molecules in the membranes doubles their bounding function with essential metabolic and bioenergetic activities.

Except for some rare authors who still envisage the cell as a naked colloid network [4], there is nowadays little disagreement that membranes are essential parts of all contemporary cells. Despite this basic acceptance concerning modern cells, we have witnessed in recent years a strong debate questioning the presence of similar membranes in the last common ancestor of living organisms, namely the cenancestor. Arguing about the presence or absence of membranes in early organisms–not only the cenancestor, but also previous organisms closer in time to the origins of life–challenges what we consider to be the basic unit of life, i.e. the cell. Unfortunately, because the lack of membranes is generally unquestioned in modern organisms, it is nowadays difficult to come across discussions about the theoretical importance of membranes.

The limited attention currently given to membranes in defining the cell concept contrasts greatly with the importance that this issue had in early cell studies. Indeed, when the Cell Theory was formulated 175 years ago in the XIX^th^ century, the reality of the membrane was unknown. Its universal character was not generally acknowledged until well into the XX^th^ century, and even when the cells were assumed to be bounded by some kind of membrane, the fluid mosaic model was not accepted until as late as the 1970s. The natural question then is: how were the cell boundaries envisioned between the formulation of the Cell Theory around 1839 and the final predominance of the fluid mosaic model in 1972? In this review, I will provide some answers to this question that I think will be useful in three different ways. First, it will considerably extend the range of some recent publications [5,6] in order to provide a more complete account of the discovery of membranes and their structure; Second, I will suggest that, contrary to the ideas favored in some articles, the discovery of biological membranes was not quite as a linear cumulative process as it has been generally depicted; And third, I expect that the acknowledgment of the importance of the cell boundary concept on modern conceptions of the cell will provide a fertile ground for discussions about the membranes in ancestral organisms. This, I hope, will open new perspectives for the stimulating field of the origins of life.

## Review

**Figure 2:**
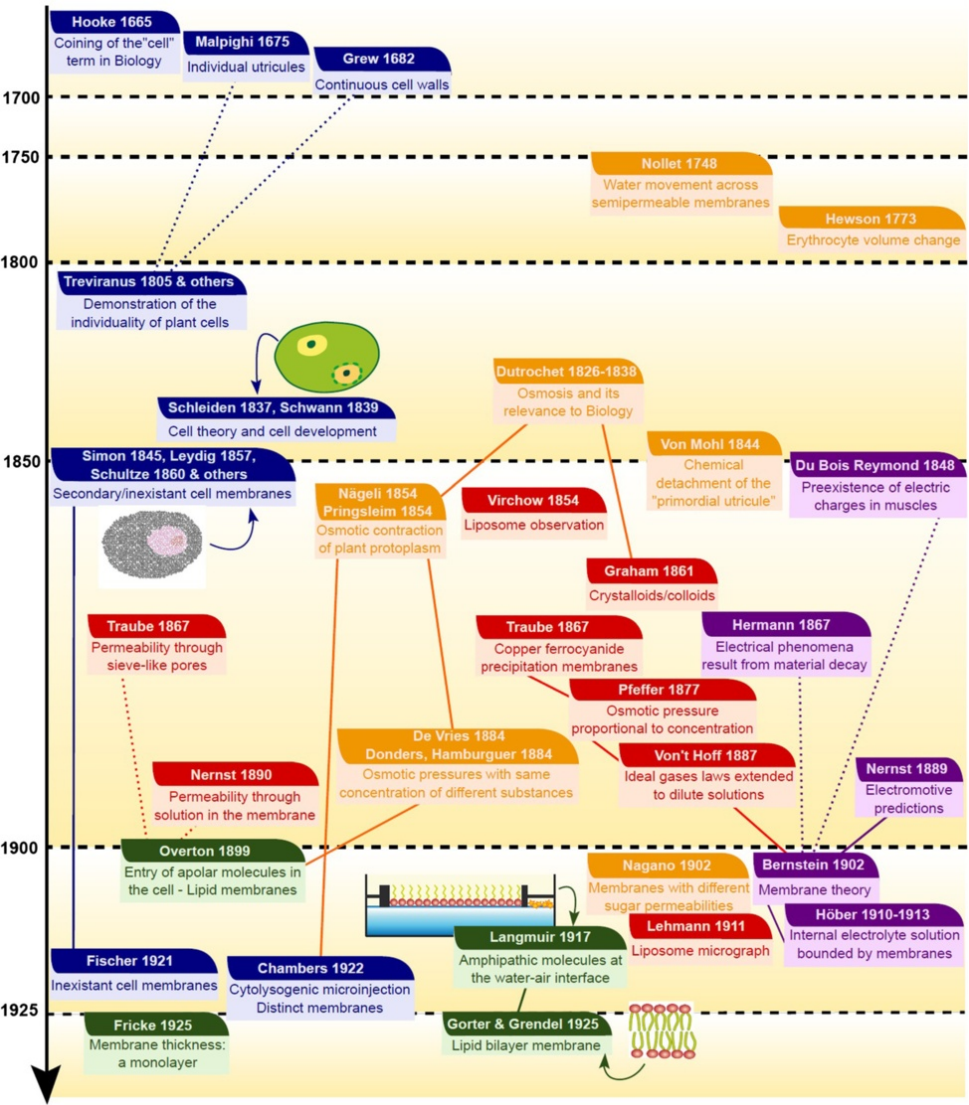
**Timeline 1665–1925.** Summary of the main contributions related to cell membrane discovery between the coining of the term “cell” in biology and the first studies on cell membrane structures. The events are approximatively ordered from top to bottom from the earlier events to the most recent. Although the studies are sometimes difficult to classify, the colors of the boxes reflect some major research axes influent to this history: **dark blue,** doubts about the existence of cell membranes; **orange,** osmotic studies; **red,** studies with artificial membranes; **purple,** electrophysiology works; **dark green,** direct description of membranes. Although most of these contributions were highly interconnected, full lines between boxes highlight particularly important relationships and dashed lines point out to contradictory views in major controversies.

**Figure 3:**
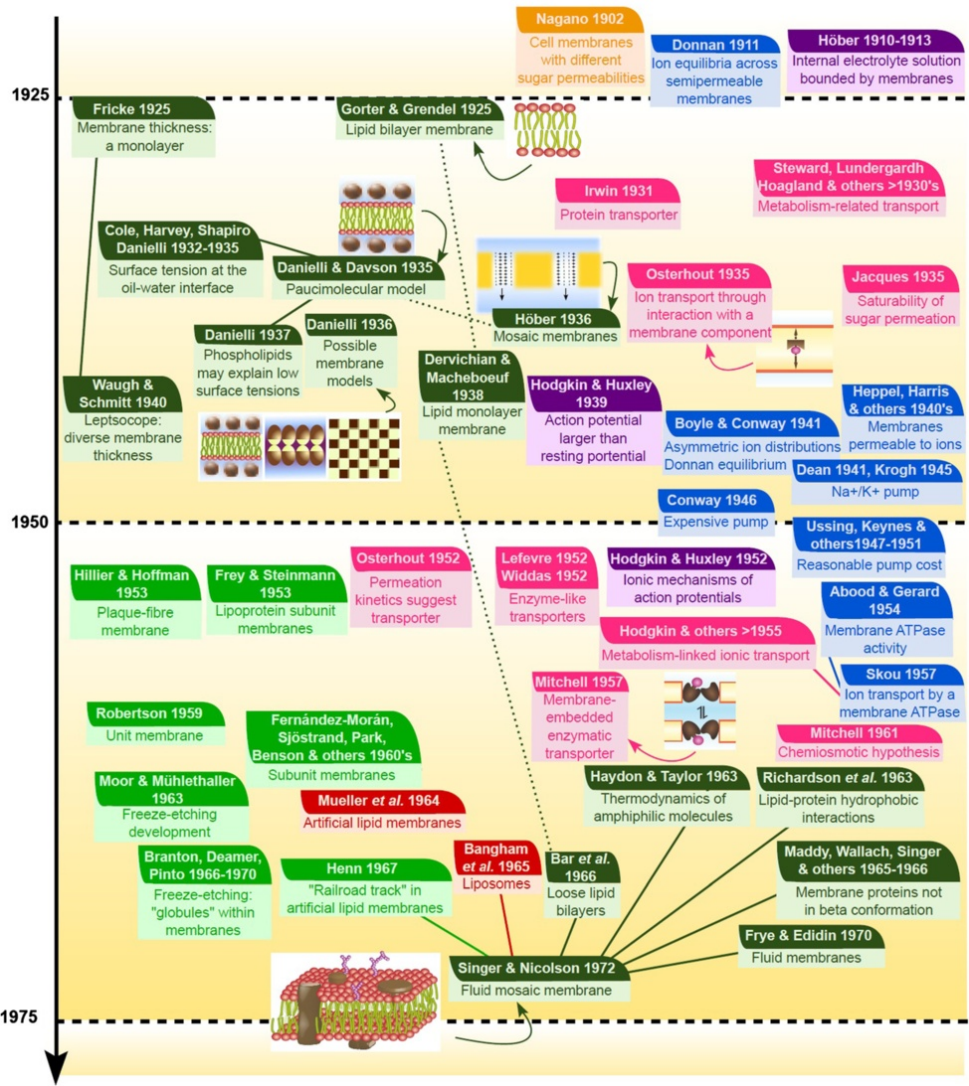
**Timeline 1925–1972.** Summary of the main contributions related to cell membrane discovery between the first studies on cell membrane structures and the formulation of the fluid mosaic model. The events are approximatively ordered from top to bottom from the earlier events to the most recent. Although the studies are sometimes difficult to classify, the colors of the boxes reflect some major research axes influent to this history: **orange,** osmotic studies; **red,** studies with artificial membranes; **purple,** electrophysiology works; **dark green,** direct description of membranes; **pink,** some transporter theories; **light blue,** asymmetric ion distribution debate; **light green,** electron microscopy studies. Although most of these contributions were highly interconnected, full lines between boxes highlight particularly important relationships and dashed lines point out to contradictory views in major controversies.

In the next sections, I will review the main discoveries that led to our current model of biological membranes: (1) the long path from the original assumptions about cell boundaries in the early Cell Theory to the first evidence that supported the existence of membranes; (2) early studies on cell membrane structure; (3) how evidence from permeability studies progressively built an alternative vision of cell boundaries–distinct from the model favored in the field of membrane studies; and (4) how the fluid mosaic model came into being. As shown in the sections below, many authors coming from different fields contributed to our understanding of membranes along the centuries. The reader will find a timeline summarizing the most dramatic contributions to membrane knowledge up to 1972 in Figures 2 and 3.

### A time before the cell membrane

It is surprising to note that most short accounts of cell membrane discovery barely discuss the early controversies on the existence of membranes around the cells [5-10]. On the one hand, most papers dealing with the characterization of membranes assume that cell membranes were a corollary of the Cell Theory. On the other hand, many authors studying the Cell Theory consider the mere osmotic or permeability studies of the late XIX^th^ century to be sufficient in extinguishing previous reluctances to the existence of membranes. In this section, we will see that the existence of plasma membranes seemed to be an unnecessary postulate for most of the XIX^th^ century. Only at the turn of the XX^th^ century, the existence of membranes became a convenient assumption for the study of most cell processes. The issue was not settled until molecular descriptions became more precise in the mid-XX^th^ century.

#### The cell walls at the time of the cell theory proposal

The construction of the cell concept was a complex process that spanned the work of a large number of naturalists from the XVII^th^ to the XIX^th^ centuries [11,12]. Since it is not my intention here to discuss the history of the Cell Theory, I will only deal with those authors whose work was particularly relevant for their conceptions of cell membranes (see dark blue boxes in Figure 2). Because many authors of this period wrote in German, the citations in the following sections will include both the primary and the secondary documents that I used to prepare this review.

In 1665, Hooke observed a piece of cork with his microscope and saw cavities that he compared to honey combs [13]. He named these cavities “cells”. This name is revealing because from the start it suggests the existence of some borders limiting an empty space. Yet, the cell boundaries that Hooke and his contemporaries could easily observe with their microscopes were the plant cell walls. Nowadays, we know that many cells can be surrounded by hard cell walls which are different from the universal cell membranes. However, since only cell walls could be easily observed at that time, early debates among microscopists focused on these structures for over 150 years.

During that period, two different conceptions of the microscopic observations competed with each other. On the one hand, some authors thought that the cell walls were continuous structures spanning the plant organism. Although he had first described the parenchyma of plants as a “mass of bubbles” [14], Grew was later the first to embrace the opinion that cell walls were made up of fibers woven together in a structure comparable to a textile fabric [15] –incidentally, giving rise to the introduction of the term “tissue” in biology [11]. This line of thought lasted until the early XIX^th^ century; its last proponent was Mirbel, who assumed that the whole plant organism was made of a unique *membranous* structure (note here that the terms “membrane” and “cell wall” were indistinctly used at that time). From his point of view, the “cells” that were observed among the “membranes” were also thought to be parts of a continuous cavity [16]. To quote one of his opponents, Mirbel’s cells were like “the bubbles in the bread crumb” [17]. On the other hand, many authors, the first of whom was Malpighi, envisioned the cells not just as the space between the “membranes” but as discrete structures bounded by cell walls [11,18]. The latter hypothesis was eventually accepted in the early XIX^th^ century when Treviranus, Moldenhawer and Dutrochet managed to separate the cells from the plant tissue using different methods [11,17,19,20]. Link’s demonstration that pigments from one cell did not pass into neighboring cells unless the cell walls were broken also contradicted Mirbel’s assumption that cavities formed a continuous compartment [11,21]. By the first quarter of the XIX^th^ century, plant cells were widely acknowledged as unconnected utricules bounded by separate cell walls [22]. Yet, the distinction between cell walls and cell membranes remained impossible.

The finding that plant cells could be separated from plant tissues contributed in shaping the increasingly popular idea that all organisms were made up of cells, namely the Cell Theory. Many biology manuals credit Schleiden and Schwann for the formulation of this theory. More thorough historical analyses actually show that the idea that cells were universal structures predated these authors and most of the features that we now recognize as cell-defining were discovered after Schleiden and Schwann [11,12]. Nevertheless, Schleiden and Schwann’s contributions were highly influential because they were among the first to intrinsically relate the idea of the universality of cells to the universality of their multiplication and growth. Their point of view on cell development deserves specific attention from us because it impacted the way people thought about cell membranes for the rest of the XIX^th^ century.

**Figure 4:**
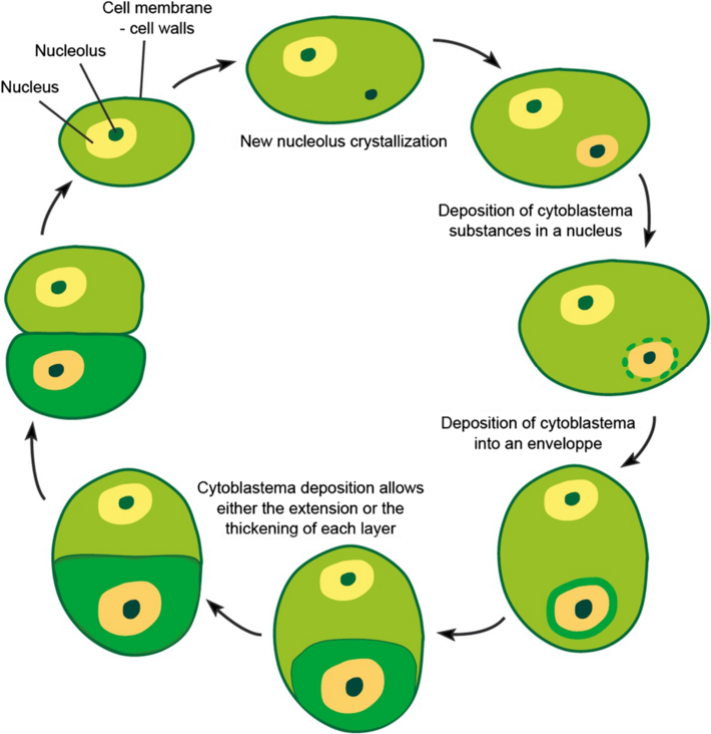
**The development of cells according to Schleiden.** This figure has been drawn for clarity from descriptions by Schleiden and Schwann, but these authors never tried to provide such a synthetic depiction in their work. Schwann’s model was very similar, except for his opinion that new cells could also crystallize from cytoblastema outside previous cells.

In 1837, Schleiden postulated a common development mechanism for all plant cells [23,24]. Two years later, in 1839, Schwann enriched and extended Schleiden’s hypothesis to animal cells, thus suggesting that there was an universal mechanism for cell development [25,26]. Their hypothesis was as follows (Figure 4): All living cells were made up of an amorphous substance called cytoblastema from which cells originated. The main difference between their respective hypotheses was that Schleiden thought that new cells always grew inside other cells, whereas Schwann acknowledged the possibility that cells could grow from any cytoblastema— whether internal or external. According to both authors, the first step for the formation of a new cell would have been the coagulation of a part of a preexisting cytoblastema into a nucleolus. The nucleolus would have acted as a nucleation center that would incorporate other molecules from the cytoblastema in a process similar to mineral crystallization. During growth, a differentiation process would have allowed the separation of the nucleus from the rest of the cell. Hardened membranes around the nucleus and the cell emerged as the result of the contact between two “phases”, i.e. the nucleus/cytoplasm or cytoplasm/environment, respectively. Although Schleiden did not discuss membranes much, Schwann considered them to be important structures responsible for separating the cell from its environment, and to be the place where “fermentation” (metabolism) took place. He assumed that membranes always limited the cells, even when they were invisible, and he suggested that the existence of membranes could be inferred from the internal Brownian movement of cell components, which did not cross the cell borders.

Despite the fact that Schleiden and Schwann’s models proved to be wrong, they acted as catalysts to foster hot debates about cell multiplication and organization that dominated histology for the rest of the century. Regarding the cell membranes, there are several points to keep in mind for the next section: First, even though Schwann assigned them essential roles, his conception of cell membranes as hardened interface structures is completely different from our current knowledge of the subject. Second, it must be recalled that, at the time, it was still impossible to make the distinction between the cell membranes and the cell wall; some people tried to look for cell walls in animal tissues and make comparisons between plant cell walls and other animal external structures, but their results were confusing [11]. Finally, many of his contemporaries called into question the assumption that, even when invisible, membranes always bounded the cells: this seemed to be theoretically unnecessary and hard to prove. As it will be developed in the next section, those who–correctly–recognized the lack of an animal equivalent to the plant cell wall predominantly assumed that membranes were not a mandatory characteristic of all cells.

#### Cells without membranes

The second half of the XIX^th^ century was a period of many fascinating biological debates and discoveries related to the Darwinian evolution, the physiology of both animal and plants and, even in histology, the discovery of mitosis. Within this context, the cell boundaries received relatively limited attention. By the early 1890s, cell membranes were often thought to be unessential secondary structures [27].

Although some authors had already thought of the cell membranes as optional secondary structures [11,28,29], it seems that the first author who explicitly dismissed the existence of cell membranes was Leydig in 1857 [11,30]. He based his opinion in the fact that membranes were not always observable and depicted the cell simply as a “substance primitively approaching a sphere in shape and containing a central body called a kernel [nucleus]” [11]. He recognized the existence of membranes as secondary structures resulting from the hardening of the cell surface. Later on, studies on amoebas reinforced the opinion that cell walls were not necessary characteristics of cells: De Bary, who understood the cell boundaries to be a solid structure like the cell walls, observed in *Plasmodium* several nuclei with no partition around each of them. He concluded that membranes may exist or not depending on different cell types [12,31]. He also reasonably argued that the presence of a rigid cell wall would have prevented the protoplasm contraction that allowed the amoeboid movements in his model organisms. In a similar way, Haeckel agreed that bounding membranes were facultative in protists [12,32].

In the second half of the XIX^th^ century, the main opponent to the existence of membranes around cells was the protistologist Max Schultze [11,12,27,33,34]. This author described the cells as small lumps of contractile protoplasm that held together because of their inability to mix with water. In his opinion, membranes were only secondary structures resulting from the hardening of the cell surface. Their appearance was an artifact that marked the beginning of the degeneracy of protists. When membranes existed, they impeded the cell division and the internal protoplasmic movements, resulting in a loss of cellular activity. Beale accordingly viewed the appearance of membranes as a mark of natural degeneracy that differentiated the active, living protoplasm from the inactive, dead material produced by the cell [35]. In 1890, Turner published a review that explored the history and updates to the Cell Theory [27]. He described plasma membranes as being secondary structures and extended this idea to their intracellular counterparts based on the fact that the nuclear membrane disappeared during mitosis. In this context, it is not surprising that Schultze and later Sachs argued for the absurdity of the very term “cell” [11,36,37]. The original term coined by Hooke stressed the existence of the cell walls, whereas these authors acknowledged the protoplasm (the protoplast, according to Hanstein, [11,38]) as the seat of biological activities.

Interestingly, although many authors from this period took for granted the absence of cell membranes, this was also the era of the first osmotic studies. Speculations about cell borders remained intact because the histological observations could not find a difference between specific cell membranes and the simple edge of the protoplasm.

#### Early osmotic studies and the cell boundaries

Osmosis studies (orange boxes in Figure 2) had an ambiguous relationship with the early understanding of cell membranes. From the earliest studies, water movement across semipermeable membranes was explicitly related to the volume changes of the cell. Osmosis can hardly be understood without the concept of membrane semipermeability and, as a result, osmotic studies have been relevant to theoretically acknowledge the cell membranes as selective barriers. Nevertheless, the first studies using artificial membranes were difficult to compare to the complexity of natural cell membranes and the analogy between the two types of membranes remained obscure for a long time.

In 1748, while he was trying to preserve some alcohol from the exposition to air, Nollet immersed a vial full of ethanol in a water container and covered it with a bladder membrane. After some hours, the bladder membrane had significantly swelled. Confronted to this observation, he carried out the opposite experiment. He put the water in the vial covered with the bladder membrane and the alcohol in the exterior container; the membrane sank. He concluded that the bladder membrane was permeable to water but not to ethanol [39]. Some years later, Hewson reported what could be considered as the first osmotic observations on living cells: He studied the shape of erythrocytes and noticed that these cells shrank or swelled depending on the salt concentration of the medium [40]. Although inspiring, these first reports went mostly unnoticed at first.

The importance of osmosis was not entirely recognized until Dutrochet’s work between 1826 and 1838. Dutrochet rediscovered the osmosis phenomena and carried out many experiments using different solutions and membranes that settled both the physical description of the phenomena and their physiological relevance. From his early works using animal membranes, he concluded that water moved from the compartment where the solutions were the less dense, acidic or positively charged to the compartment where the substances were more dense, alkaline or negatively charged [41]. He was influenced by previous work by Porret, who showed that an otherwise impermeable animal membrane could become water-permeable when an electric current was applied [42]. Dutrochet repeated Porret’s experiments and first thought that the water movement across the membrane could be somehow related to electricity [41]. His contemporaries also suggested that capillarity or viscosity differences between the solutions may account for the observed phenomena [43,44]. However, a more systematic analysis allowed Dutrochet to discard all these hypotheses, including his own [45]. He concluded that the reason for the water movement was the heterogeneity of the liquids in the two compartments, but the underlying nature of the heterogeneity remained unknown to him [44]. It may be surprising to notice how hard it was for Dutrochet to explain the phenomena that he observed, but this has to be considered in its context: Diffusion was qualitatively described by Graham in the 1830s and Fick did not provide his quantitative equations for diffusion until 1855 [46,47].

From the beginning of his experiments, Dutrochet extended his observations on osmosis to physiology [41]. He explained plant turgescence by the fact that plant cells used osmosis to accumulate water. His work on osmosis certainly influenced his opinion that cells were surrounded by essential cell membranes, though his way of describing them looks alien to us today. He suggested that cell borders acted as “chemical sieves”, which we would now describe as semipermeable, although he did not use that term. The chemical sieve-membrane would have been able to change the composition of the cell medium, resulting in the “secretion” (~metabolism) of substances to both the exterior and the interior of the cell [41].

#### Later osmotic studies and artificial membranes

In 1844, Von Mohl treated plant tissues with alcohol and different acids and described the detachment of the protoplasm from the interior of the cell walls. He named the shrinking vesicle that separated from the cell walls the “primordial utricule” [11,48-50]. In addition to this chemical method, in the 1850s Nägeli and other authors put plant cells in hypertonic media and observed the contraction of a vesicle within the cell walls [11,12,51-54]. Over time, such osmotic studies became more quantitative, and by 1884, De Vries and Hamburguer among other authors were able to use plant and animal cell models to show that, except for electrolytes, most solutions applied equal osmotic pressures at equal concentrations [55,56].

Although today these results may seem quite straightforward to analyze, at their time they were not decisive because the membranes remained invisible. It is unclear if Von Mohl thought that the primordial utricule was surrounded by an envelope or was a naked portion of protoplasm [11]. Nägeli believed that the semipermeable membrane resulted from the hardening of the exterior layer of protoplasm in contact with water, thus supporting the idea that membranes were not different from the rest of the cell [54]. Jacobs has argued that, even after their detailed osmotic studies, de Vries and Hamburguer did not assume that cells were necessarily bounded by membranes [57]; de Vries was actually aware of the fact that the osmotic phenomena he was measuring reflected volume changes in the massive plant vacuole, thus diverting the attention from membranes [11,55]. Finally, the opponents to cell membranes were comforted by Nägeli’s experiments on the idea that the naked protoplasm was the active component of the cell, whereas the rigid cell wall was an unessential secondary element that could even be removed from the cell [27].

Two concepts are important here to understand how the cell was portrayed in the late XIX^th^ century and early XX^th^ century: the colloid and the precipitation membranes (red boxes in Figure 2). In 1861, Graham separated the water-soluble molecules into two sorts according to their ability to cross a parchment paper: Inorganic salts and sugars easily crossed the membrane and were called crystalloids, whereas gelatin-like compounds were unable to do so and were named colloids [58]. Biologists rapidly adopted the term “colloid” to refer to the structure of the protoplasm, probably because it seemed more precise in describing the viscous jelly-like interior of the cell. The open question then was to determine if the surface of the cell was made up of the same substance as the rest of the protoplasm. A possible answer to that question was provided by studies on precipitation membranes.

**Figure 5:**
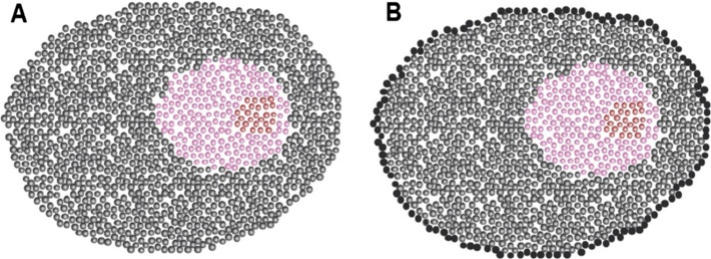
**XIX** ^**th**^ **century doubts about the existence of membranes. A**. In this vision, the cell is devoid of any membrane and all the properties of the cell are defined by the activity of the protoplasmic colloid. **B**. The cell is surrounded by an external layer (membrane) of which the nature is distinct to the rest of the protoplasm. Yet, in this view, the inside of the cell remains a colloid.

Precipitation membranes were the first artificial membranes to be synthesized. They were first developed by Traube in 1867 and so named because they were obtained by the precipitation of molecules at the interface between one solution of potassium ferrocyanide and another of copper sulfate [59,60]. These membranes were permeable to water but not to other molecules, thus becoming important tools for osmotic studies. For instance, Pfeffer was able to produce sturdier precipitation membranes in 1877 and carried out several experiments that established the correlation between the osmotic pressure and the solution concentration and temperature [60,61]. Precipitation membranes were highly influential in the debate about the existence of cell membranes because most authors, including those working on osmosis, assimilated the cell membranes to a precipitation membrane. According to that point of view, the protoplasmic colloid precipitated when it was in contact with the aqueous medium, but this did not require the membrane to be any different from the rest of the protoplasm (Figure 5).

To summarize, despite the important developments in osmosis that were taking place during the second half of the XIX^th^ century and the intrinsic necessity of membranes to explain this phenomenon, it would be misleading to think that cell membranes were considered mandatory cell structures at the time. Even those who acknowledged the fact that osmosis requires semipermeable membranes to take place, envisioned cell borders as precipitation membranes at the surface of a protoplasmic colloid–a point of view irreconcilable with our current understanding of cells.

#### Late membrane-less hypotheses

As it has been shown so far, during most of the XIX^th^ century, cell membranes attracted limited attention and the dominant opinion was that cell membranes (often mistaken for cell walls) were secondary structures that resulted from the contact between the protoplasmic colloid and the environment. This vision of things changed at the turn of the XX^th^ century but marginally remained until well into that century. In this regard, Fischer’s work in 1921 is noteworthy because he called into question the concept of cell membranes at a time when their existence was generally taken for granted but direct evidence remained scarce. His arguments, which can be considered as the culmination of the XIX^th^ century point of view on membranes, were the following [62]: 1) Cell membranes were invisible using optical microscopy; even when the edge of the cell was visible, it did not prove the existence of membranes with characteristics different from the rest of the protoplasm; 2) When cells were immersed in a hypertonic medium, they shrank less than would be expected from strict osmotic criteria; 3) Cell permeability to different molecules seemed to change depending on many factors—an observation seemingly incompatible with contemporary descriptions of the membrane as sieves or apolar solvent layers; 4) Fischer claimed that cell fragments behaved similarly with solutes than whole cells, although he did not provide a precise account of the experiments that made him say so; and 5) Since he assumed that the interior of the cell was a colloid, he argued that cell membrane models still had to explain how the molecules moved within the colloid (the cell) once they had crossed the membrane. As a result of all these criticisms, he concluded that the external layer of the protoplasm could only be conceived as a “surface tension film” made up of the same compounds as the protoplasm and lacking any osmotic value.

Fischer’s opinions were certainly not dominant at the time, but the colloid hypothesis did not die out until progress in enzymology and molecular biology replaced the homogeneous, gelatin-like description of the protoplasm by studying discrete compounds of life. The existence of membranes was vindicated as late as the 1950s and there are even some authors who still call it into question [4,63].

#### The birth of cell membranes

The existence of cell membranes did not become popular until the turn of the XX^th^ century. In this section I will present the different pieces of evidence that led to the general acknowledgement of the existence of cell membranes in its modern sense.

##### The permeability of molecules according to their polarity

In the 1890s, two confronting views competed each other to explain how semipermeable membranes operated: Traube had suggested that precipitation membranes had small pores that allowed them to behave like sieves, whereas Nernst introduced the idea that permeating substances were those that could dissolve in the membranes [59,64,65]. From 1895 to 1899, Overton carried out a series of experiments in which he immersed cells in solutions of over 500 different substances at the same concentration in order to study their permeability with different molecules [66-68]. He noticed that solutions of ether-soluble (apolar) molecules did not result in the shrinking of cells, contrary to solutions of water-soluble (polar) substances. He concluded that apolar molecules entered the cells with less difficulty than polar substances, and he showed that this was irrespective of their molecular size. Since solubility, not molecular size, was the best predictor of the entry of substances in the cell, Overton favored Nernst’s hypothesis for membrane permeability [68]. Based on the observation that not all molecules could enter the cells with the same ease, but also aware that the cellulose cell wall could not be involved in the phenomenon, he suggested that there was a cell membrane distinct from the cellulose cell wall [11,66] and that these cell membranes were made up of ether-soluble components [67,68]. Looking for specific polar candidates that could make up the membranes, he ruled out the triglycerides because they would be subject to saponification in the regular living conditions of cells. He suggested that cholesterol and phospholipids could be the main components of cell membranes even though little was known at the time about the cellular functions of these molecules. He also recognized the difficulty that the membrane solubility theory may introduce in explaining the movement of water and other hydrophilic substances across the cell boundaries. He tried to solve this paradox by recalling that, in spite of their hydrophobic nature, cholesterol esters and cholesterol-lecithin mixtures were known to absorb large water volumes [68]. He also suggested that some kind of active property of the protoplasm could allow the active transport of molecules into the cell [66,69]. Similar, lesser-known observations were also reported in bacteria and analyzed in a similar way [70,71]. Yet, Overton remained the authoritative figure to which most future cell membrane works would refer (dark green boxes in Figure 2).

##### The permeability to dyes

In 1855, Nägeli had already made some interesting observations about the permeability of dyes in the plant cell [52,54]. First, he noticed that when plant vacuoles were filled with a pigmented solution, the osmotic changes could modify the volume of the vacuole but the pigment did not leak outside the vacuole unless it was artificially damaged. He also showed that when plant cells were immersed in hypertonic colored solutions, the protoplasm shrank and the colored solutions could be observed in the space between the protoplasm and the cell wall, but the pigments did not enter the protoplasm. As a result of both observations, he concluded that the vacuole boundaries and the protoplasmic surface were barriers to penetration [52,54]. Still, as we have seen previously, Nägeli did not think that the cell was bounded by a differentiated membrane so he assumed that the resistance to pigmentation was a general characteristic of the whole protoplasm rather than the consequence of the activity from a specific part of the cell. Overton revived the interest for dyes and, in accordance with his previous work, visually confirmed that lipid-soluble dyes entered the cells more easily than water-soluble dyes [11,72]. Yet, these observations did not definitively prove that cell membranes were chemically different from the rest of the protoplasm. It was not until 1922 that the improvement in the microinjection techniques provided a crucial answer to this issue. Chambers used this technique to apply a water-soluble cytolysogenic (i.e. able to digest the cytoplasm) solution in different parts of the cell. He showed that he could apply the hydrophilic cytolysogenic substance on the surface of starfish eggs without damaging them. Then, he injected a small amount in the interior of the cell and he observed the cytolysis of the protoplasm. When the injection was made close to the cell borders, the cytolysis spread in the protoplasm but did not impact the membrane until the rest of the cell had been massively damaged [73]. This was the first unavoidable evidence that the nature of the cell surface was different from the rest of the protoplasm, supporting the existence of the cell membrane.

##### The electrophysiology of excitable cells

The electrophysiology is a domain unto itself, so here I will only briefly summarize the main indirect contributions to the field of cell membranes at the turn of the XX^th^ century (purple boxes in Figure 2). There are two components in this story. The first is the primal electrophysiology debate incarnated in the opposition between du Bois-Reymond and Hermann [74]: In 1848, the former had reported an electric current and action potential in muscles and nerves and tried to explain them by the preexisting charge differences between the interior and the exterior of the tissues [74,75]; In 1867, the latter assumed that the currents measured by du Bois-Reymond were an artifact and that the electrolytes found in the external medium resulted from the chemical decomposition of the samples [74,76]. The second element of this story is the field of electrochemistry, which was emerging very fast thanks to the development of more precise mathematical models. It began when de Vries attracted Von’t Hoff’s attention to the problem of osmotic pressure. Von’t Hoff first used the osmotic measurements from Pfeffer, De Vries, Hamburguer, Donders and Raoult to suggest the theoretical and experimental equivalence between the laws of ideal gases and those that ruled the behavior of dilute solutions [77,78]. The main exception that did not seem to fit into Von’t Hoff’s hypothesis were electrolytes. Arrhenius contacted Von’t Hoff in a personal communication and put him on the track to explain the observed anomalies based on the dissociation hypothesis. Eventually, Nernst, who was studying the relationship between electricity and electrolyte movements, developed Von’t Hoff’s equations for the calculation of the electric potential and electromotive force in galvanic cells [79,80].

These two lines of research met through Bernstein’s work in 1902. Bernstein used a physiological model to corroborate some of the electrochemical predictions made by Nernst. In particular, he showed that temperature changes impacted the electromotive forces in muscles according to Nernst’s predictions [81,82]. The reconciliation of electrophysiology with electrochemistry allowed Bernstein to formulate the “membrane theory of electrical potentials”. Bernstein’s membrane theory postulated that (1) nerves consisted of a conducting electrolyte bounded by thin membranes impermeable to ions; (2) in the resting state, the membrane kept an electric potential with internal negative charges and external positive charges; and (3) in the activity period, potassium permeability increased and the electric potential consequently dropped [81,82].

In the 1910s, Höber carried out a series of experiments that corroborated Bernstein’s theory and coincidentally provided supplementary evidence in favor of the existence of cell membranes. Höber showed that conductivities of muscle or compacted erythrocytes were higher at high electrical frequencies than at low frequencies. He suggested that membranes were impermeable at low electrical frequencies but became less resistant at high frequencies because the cells themselves were disrupted. In order to test this hypothesis, Höber measured the internal conductivity of the cells. The values that he obtained were not compatible with the attachment of electrolytes to the protoplasmic colloid, but they supported their solution in the internal medium. The corollary of these results was that the only way to prevent the electrolytes from diffusing out of the cell was to present an impermeable boundary with properties different from the rest of the protoplasm [83-86].

##### The existence of cell membranes

In short, in the early XX^th^ century the presence of membranes was becoming widely accepted and was supported by three lines of evidence: Overton’s permeability studies, Chamber’s microinjection experiments and Höber’s electrical measures. Nonetheless, it should be noted that for some organisms, especially bacteria, this debate was not definitively closed until several decades later, when cell membranes could be directly observed using the electron microscope [87-89].

### The first membrane structures

In the previous section, I have shown that many authors in the XIX^th^ century thought that membranes were not essential parts of cells and even those who recognized their importance did not conceive membranes as we do today. By the turn of the XX^th^ century, cell membranes had become a convenient assumption supported by a few direct experiments. The next decades would witness an increasing interest in describing membrane structure (dark green boxes in Figure 3).

#### Gorter and Grendel: a relative breakthrough

In the early XX^th^ century it appeared clear that, if cell membranes existed, they would likely be at least partially lipid-based. The opinion that the cell surface could be covered by a thin lipid layer goes back to the 1880s [65,90] but it was not popularized until Overton’s publications in 1895–1899 (see previous section). The molecular structure of membranes remained unexplored until the major breakthrough made by Gorter and Grendel in 1925.

**Figure 6:**
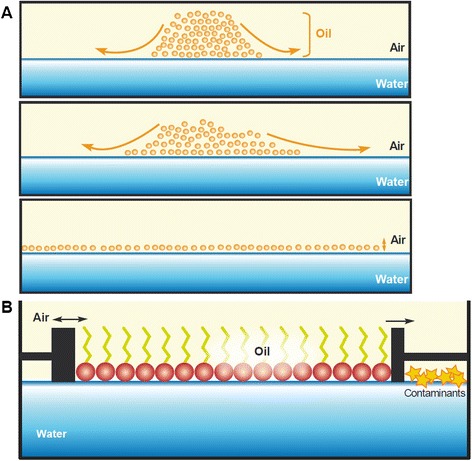
**Oil at air/water interfaces. A**. Oil molecules spontaneously spread on the air/water interface until they form a layer one molecule thick. **B**. The Langmuir trough allows to precisely measure the surface that these monolayers can spread depending on the applied pressure.

**Figure 7:**
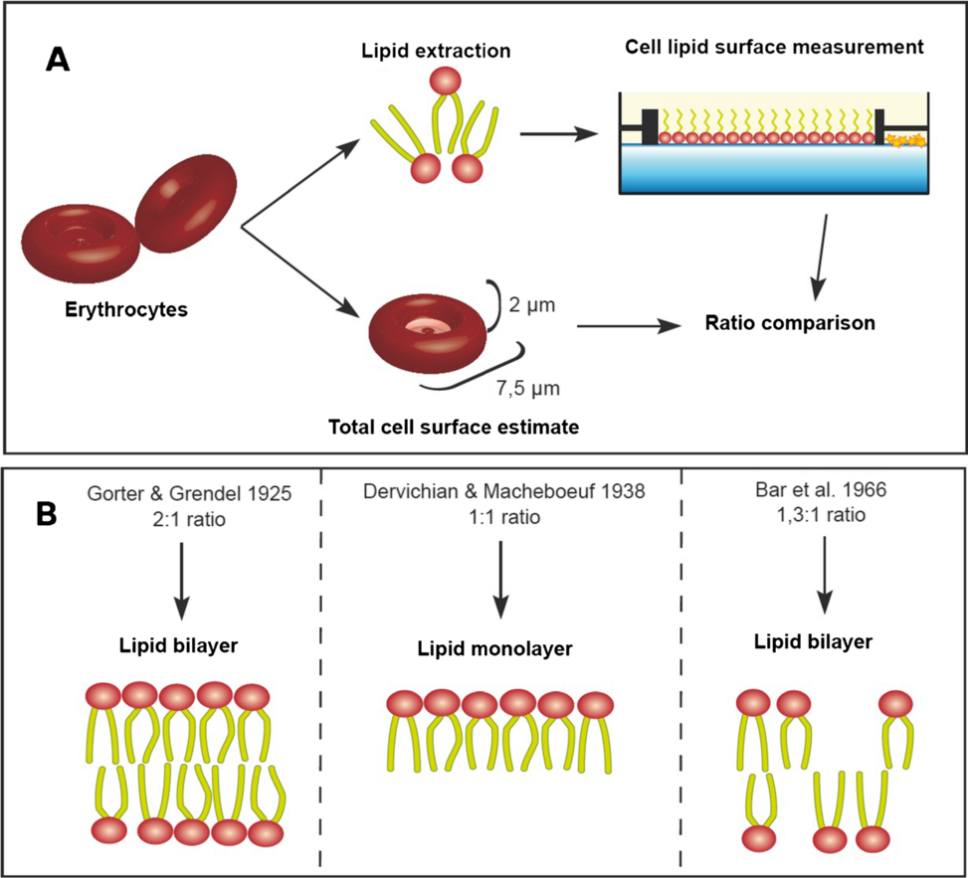
**Surface measurement of membrane lipid monolayers as a way to determine membrane structure. A**. Summary of the method, consisting in the comparison between the surface occupied by lipids extracted from membranes and the estimated surface of cells **B**. Different results and interpretations.

The genius of this paper was to compare the surface that cell lipids were able to occupy to the total surface of cells. Gorter and Grendel extracted the lipids from an erythrocyte sample; since these cells were known to lack internal membranes, they assumed that all lipids should come from the cell envelopes. Measuring the spread of lipids on water was done using a Langmuir’s trough (Figure 6). This device had first been developed by Pockels as a way to precisely measure the surface covered by lipid monolayers at the interface between water and air [91], but it was named after Langmuir’s version more than 25 years later [92]. When Gorter and Grendel compared the surface covered by the lipids to the estimated sum of cell surfaces, they found a 2:1 ratio (Figure 7). As a result, they concluded that cells were surrounded by a lipid membrane two molecules thick–a lipid bilayer–with the hydrophobic components in the internal part of the membrane and the hydrophilic components in the external part [93].

This study has been commonly cited as the most conclusive argument in favor of the lipid bilayer nature of cell membranes. In spite of the elegance of this work, it is important to balance its contribution to the field because it is subject to criticisms from both technical and theoretical grounds. First, the extraction technique employed could not isolate the totality of erythrocyte lipids from the samples [94]. In addition, the equation that Gorter and Grendel used to estimate the surface of the erythrocytes also underestimated the cell area [94]. Some historical reviews on membrane discovery have argued that it was fortunate that the two errors neutralized each other in order to give credit to the lipid bilayer hypothesis that we now recognize in current membrane models [5]. But it should be recalled that the lipid bilayer hypothesis of 1925 did not leave room for anything else than lipids to be located in the membrane plane–in contrast to past and current mosaic hypotheses. Some parallel reports even contradicted Gorter and Grendel’s values (Figure 7). Dervichian and Macheboeuf carried out a similar analysis and obtained a 1:1 ratio; as a result, they assumed that the cell membrane was a lipid monolayer [95]. Although this second work also had lipid extraction problems, the major difference between the two studies was that Gorter and Grendel measured the surface covered by lipids at the first detected pressure (i.e. the maximal continuous surface covered by an amount of lipid) whereas Dervichian and Machebouef measured the surface right before the collapse pressure (i.e. the minimal surface before the monolayer collapsed). In the 1960s, a more accurate ratio for the lipid surface at the collapse pressure with respect to the cell surface estimates was calculated to be 1,3:1 [94]; the authors of this later study suggested that their ratio conformed to loose lipid bilayers, whereas the modern interpretation of the fluid mosaic model supports the idea that membrane lipids are tightly packed and the “excess space” is actually occupied by membrane proteins.

Apart from the technical issues, it is worth noting the theoretical bases on which Gorter and Grendel founded their lipid bilayer concept. Some years earlier, in the 1910s, some pioneer papers had studied the behavior of amphiphilic molecules at the interface between water and air [92,96,97]. For instance, in 1917 Langmuir used the valence bond theory suggested by Lewis the previous year [98] to explain molecular hydrophily on the grounds of “secondary valence”, i.e. chemical polarity [92]. Langmuir put forward this hypothesis in the same paper in which he presented the trough that, somewhat unfairly, was named after him. Notwithstanding, Gorter and Grendel only cited Langmuir’s paper in a very superficial way related to the trough [93]. Instead of reasoning in terms of hydrophily and hydrophobicity, they suggested the bilayer structure based on crystallographic studies [99] and soap bubble observations, which were only distantly related to their subject [100]. Gorter and Grendel are not really to blame because hydrophobic interactions were very poorly understood at the time. Subsequent authors, like Danielli, discussed the importance of the amphipathic nature of lipids and proteins to account for their respective structural hypotheses, but they disregarded the importance of hydrophobic interactions [101]. Even Langmuir, who extended his explanation of amphipatic molecules to protein monolayers [102], overlooked the hydrophobic interactions between proteins and lipids [103] when he came to envision the cell membrane.

In summary, although Gorter and Grendel’s work was decisive for making the lipid bilayer concept popular in 1925, its actual contribution to current membrane models can only be appreciated in the light of later progress in membrane studies and hydrophobic interaction understanding [6].

#### First direct studies on membrane structure

The formulation of the lipid bilayer hypothesis had opened the door to the molecular description of cell membrane structure. In an attempt to confirm or refute the lipid bilayer postulate, one of the first lines of research to be explored was the measurement of membrane thickness. The first attempt to estimate the thickness of cell membranes was directly related to Höber’s research on cell conductivity (see above). In 1925, Fricke measured the static capacitance per surface unit with an estimation of the cell membrane dielectric constant. He extrapolated that the thickness of erythrocyte and yeast cell membranes was in a range between 3.3 and 4 nm [104,105]. This thickness was compatible with a lipid monolayer but not with a bilayer, thus providing support to the monolayer membrane proponents [95]. The choice of the dielectric constant used in these studies was called into question but the subsequent tests could not refute the estimation by Fricke [106]. Independently, the leptoscope was invented in order to measure very thin membranes by comparing the intensity of light reflected from a sample to the intensity of a membrane standard of known thickness [107]. This device measured thicknesses that depended on pH and the presence of membrane proteins and ranged from 8.6 to 23.2 nm. As a result, the lower values supported the lipid bilayer hypothesis whereas the higher ones could support the presence of supplementary superimposed layers [108]. The thickness of the membrane would only become really accessible two decades later, when the observation of membrane sections using electron microscopy established the now accepted ~8 nm value for standard cell membranes [109].

**Figure 8:**
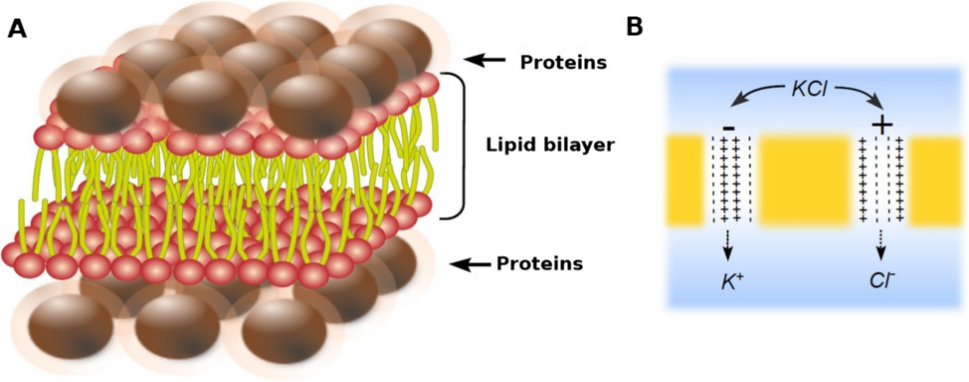
**Membrane structure hypotheses in the 1930’s. A**. Paucimolecular model, with a lipid bilayer coated with proteins in both sides. **B**. Höber’s mosaic model in which membranes behaved both as solvents and sieves.

The 1930s were important in this field because the decade introduced the most influential membrane structure model until the general agreement on the fluid mosaic model, namely the paucimolecular model (Figure 8). The genesis of this model relied on studies of surface tension between oils and echinoderm/teleostei eggs [110-112]. As the surface tension values appeared to be much lower than would be expected for an oil–water interface, it was assumed that some substance was responsible for lowering the interfacial tensions in the surface of cells [112]. At this time, membranes were known to contain substantial quantities of proteins [113,114] but little had been said about their position in cell membranes. Therefore, in 1935 Danielli and Davson suggested that the lipid bilayer was sandwiched between two thin protein layers [115]. Although the paucimolecular model was characterized by the superposition of protein and lipid layers (Figure 8), the authors were aware of the contemporary debates about membrane permeability (discussed later), so they admitted the possibility that some proteins could span the membrane.

The paucimolecular model immediately became popular and it dominated cell membrane studies for the following 30 years. From the beginning, it was confronted with a balance of supportive and critical observations. Among the so-called supportive evidence, there were some light polarization and X-ray diffraction studies. In order to provide insights on membrane structure, these methods required repeated structures, so the samples used were myelinized axons. These analyses showed an alternation of protein and lipid layers in support of the paucimolecular model [116-118]. Unfortunately, we know now that this biological material is physiologically very specific and can hardly be compared to a regular cell membrane.

The hypotheses that challenged the paucimolecular model will be further developed below, but first I will present some details regarding the aforementioned studies on surface tension. The surface tension experiments that led to the paucimolecular model used triglyceride oils and other non-miscible lipids. These substances are appreciably different from most natural amphipathic cell membrane components and, therefore, they are not suitable for a realistic comparison to cell membranes. Danielli and other authors showed soon after the postulation of the paucimolecular model that the addition of amphipatic cell membrane components like fatty acids, cholesterol or phospholipids to non-miscible mixtures was very effective in dropping the interfacial tension between water and highly hydrophobic substances [119,120]. As a result, shortly after the suggestion of the paucimolecular model, the main argument that motivated it, namely the requirement of proteins to lower the superficial tension of the cell, had been dismissed. Yet, the hypothesis remained popular for 30 more years.

#### Contemporary competitors: the mosaic models

Despite of the prevalence of the paucimolecular model in the mid-XX^th^ century, this hypothesis was not devoid of competitors. Direct experiments on cell surfaces were scarce but permeability studies provided an outstanding playground for indirect speculations about cell membrane structure. Permeation studies suggested what would to be called the “mosaic models”.

As it was previously pointed out, Overton’s hypothesis that apolar molecules easily entered the cell because they could get dissolved in the cell lipid membranes automatically raised the problem of how polar molecules accessed it. In an attempt to circumvent this issue, Nathansohn suggested in 1904 that the cell surface could be a mosaic combining fat-like parts with protoplasmic-like parts [62,121]. In the early XX^th^ century, the “mosaic” term was recycled to refer to membranes with heterogeneous parts [122-124]. In the 1930s Höber amended it to fit the idea that membranes were a mixture of sieve-like and solvent (i.e. lipid) parts [125-128]. These mosaic hypotheses were the result of the combination of the two classic ways of understanding the permeation through membranes: Traube’s precipitation membranes and Nernst/Overton lipid membranes. In the first case the molecular and pore sizes were predicted to best account for molecule transport; in the second, the hydrophobicity was the best predictor of molecular permeability. The compromise reached described the cell membranes as lipid layers interrupted by pores (Figure 8, [126]). Since the permeability of some polar molecules had been shown to change according to different conditions [129,130], it was also suggested that the pore diameter could change according to the hydration of the pore, the pH, its obstruction by some particular molecule, the membrane stretching, the metabolic activity and the cell type [126]. Most descriptions did not specify the type of molecules that could form these pores, but proteins were among the best candidates [101,131].

Apart from the mosaic hypotheses proponents, other authors also called into question the paucimolecular model. For example, in their presentation to the very influential 1940 symposium on the permeability of cell membranes, Parpart and Dziemian reported the chemical composition that could be analyzed from cell extracts [132]. Although these authors did not specifically support the mosaic models, they noted that lipases in contact with cells modified the cell permeability, which suggested that surface phospholipids were naked instead of coated with proteins. The discussion that followed their talk is remarkable because several authors exposed their visions for membrane protein structure: Ponder imagined the proteins adopted a spaghetti-like shape on top of the membrane whereas Davson suggested that proteins could span the whole erythrocyte, not just the membrane.

**Figure 9:**
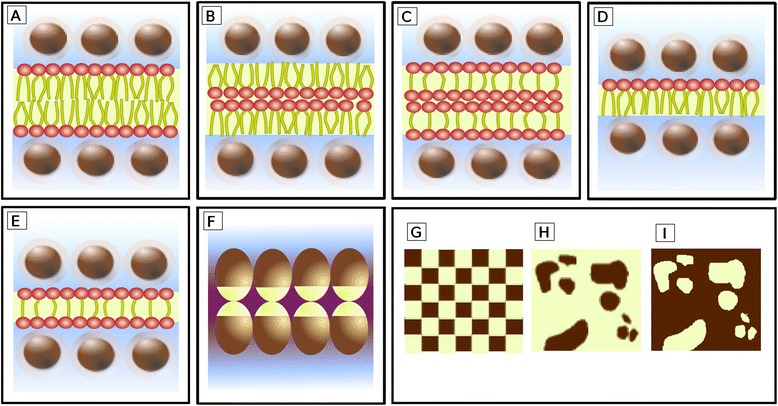
**Possible molecular arrangements of biological membranes redrawn from Danielli in 1936** **[** 101 **]** **. A-E**. Cross-section of hypothetical membranes with internal lipids and coating proteins. **F**. Cross-section of an hypothetical membrane made up of lipoprotein subunits. **G-I**. Surface of mosaic membranes.

In 1936, Danielli, who was probably the most influential author in the field at the time, discussed a complete catalog of possible membrane structures in addition to his paucimolecular model [101]. He excluded all membrane models that were much thicker than 8 nm because he thought it was the most plausible cell membrane thickness. He classified the membrane models in three types: continuous lipid membranes, mosaic membranes and lipo-protein membranes (Figure 9). In the first type, he imagined all the possible combinations of lipid monolayers and bilayers coated with proteins. He concluded that a lipid bilayer with the polar parts of the lipids in the exterior would be the most stable structure because it maximized the contact of the hydrophilic lipid parts with water. He assumed that the proteins were subject to the same amphipatic constraints as lipids but in their own layers. In the mosaic-like models, he considered different distributions of proteins and lipids, but he ruled all of them out because he assumed that lateral interactions between lipid hydrophobic parts and proteins would not have been stable. He also considered that if the lipid bilayer had not been covered by proteins, it would not have been solid enough to provide a reliable impermeable barrier to resist to cell deformation. Finally, he did not go into the detail of the lipoprotein membranes because little was known about such kind of molecules. Concerning ion permeability, he acknowledged the three popular possibilities of his time: pores, simple diffusion and the existence of some kind of transporter in the membrane [133].

In summary, many hypotheses on cell membrane structure were under discussion in the late 1930s. Among these, the paucimolecular and the mosaic models were certainly the most iconic. The next developments would be highly influenced by the evidence from independent, though membrane-related, fields.

### Insights from the movements across the membrane

The direct characterization of membrane structure did not progress much until new techniques allowed dramatic discoveries in the late 1950s and 1960s. Before these new methods became available, cell membranes attracted the attention from authors who were studying membrane roles in physiology and metabolism. Although these contributions were not always immediately recognized by the community working on membrane studies, it is important to take them into account because they illustrate how works of this period (1940s-1950s) indirectly changed membrane understanding.

Most of the studies presented in this section are related to molecular transport across membranes and did not directly address the question of membrane structure. Therefore, I will not go into detail for discoveries in these fields but I will just provide some general clues to illustrate how the contextual research impacted cell membrane conceptions. For more complete accounts on the history of transport, excitability and membrane metabolism I invite the interested reader to refer to the insightful works by Robinson and Kleinzeller and colleagues [134,135].

#### Asymmetric ion distributions

In the early XX^th^ century, Na^+^, K^+^ and other ion concentrations were already known to be different between the interior of the cell and their environment [136,137]. Three classic hypotheses competed to explain these observations: (1) some ions were stably bound to the cell colloid; (2) the membranes were totally impermeable for some ions; and (3) ion concentrations were kept at the expense of an energy-consuming transport [134,138].

The first hypothesis is by definition related to the attachment or dissolution of molecules in the protoplasmic colloid. As we have seen previously, the electric measures carried out by Höber and Fricke suggested that the cell internal medium could be compared to a conductor solution of free electrolytes [85,86,104]. References to the so-called interactions between ions and the colloid lasted for some decades [139,140], but the colloid concept progressively became outdated with the developments of enzymology and molecular biology [63]. As a result, ion asymmetry was mainly debated as an opposition between the two other possibilities: membrane impermeability or energy consumption (light blue boxes in Figure 3).

**Figure 10:**
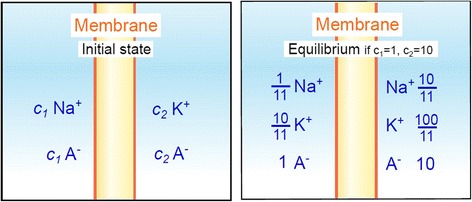
**Donnan’s equilibrium.** Two solutions containing two different initial concentrations of different salts are separated by a membrane. In this case, the membrane is impermeable to anions but permeable to cations. Donnan thermodynamic calculations and experiments showed that, contrary to what could be initially thought, the two cations do not just interchange with each other until they are equally distributed in the two compartments. Instead, equivalent quantities of both cations cross the membrane; as their initial concentrations are different, the cation which was initially less concentrated proportionally crosses the membrane more than the initially highly concentrated cation.

In 1910, Donnan had shown that, provided a membrane was permeable to some ions and impermeable to others, an ion distribution asymmetry was expected to spontaneously arise according to the second law of thermodynamics (Figure 10, [141]). In 1941, Conway and Boyle suggested that the membrane impermeability to negatively-charged proteins and one or several electrolytes could generate a complicated Donnan effect that would account for the observed ion asymmetry [142]. Nevertheless, the simultaneous accumulation of evidence–especially from diet studies and radioactive cation labeling–supported the hypothesis that physiological ions could effectively cross the membranes [143-147]. Moreover, the increased interest in blood conservation during the Second World War demonstrated that K^+^ loss from erythrocytes was related to the slowdown of metabolism [148,149]. Finally, cation fluxes seemed able to restore ion asymmetries in the recovery period after muscle stimulation [150].

This evidence led Dean to postulate in 1941 that the agent of ion movement against the gradient could result from the activity of some “pump” located in the muscle fiber membrane [151]. The Croonian lecture by Krogh in 1945 was also an influential opinion in favor of the hypothesis that Na^+^/K^+^ asymmetries were the result of active transport [152]. Conway replied to this hypothesis by attacking some aspects of the experimental designs and interpretations of his opponents (see [134] for details), but his most commented objection was that his own calculations on the energy necessary to extrude all the Na^+^ from the muscle was larger than the actual available energy in the resting muscle [153]. He admitted that some small amount of Na^+^ transport could be possible, but in derisory proportions. Nonetheless, the impermeability hypothesis was progressively abandoned when new works measured the outflow of radioactive cations more precisely and calculated a more reasonable amount of energy to account for the active cation transport–the active transport hypothesis became predominant [154-157].

**Figure 11:**
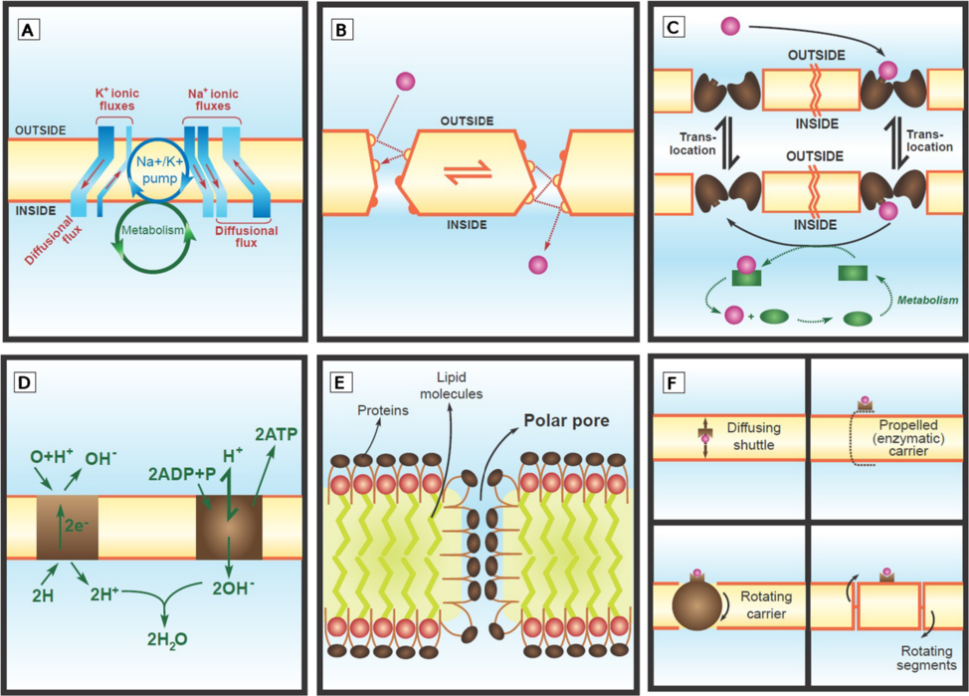
**Redrawings of some examples of transport across membranes in the 1950’s and 1960’s. A**. Eccles depicts in 1963 the coupling between a metabolic-driven ion pump and several different channels [165]. **B**. Burgen suggests in 1957 that molecules cross pores thanks to specific and dynamic interactions with them [166]. **C**. Mitchell describes in 1957 an enzymatic-like protein transporter embedded in the membrane [167]. **D**. Mitchell’s chemiosmotic hypothesis in 1961 is based in the existence of structures embedded in the biological membranes [168]. **E**. From an early date, Danielli and collaborators considered the possibility that channels may have existed within their paucimolecular hypothesis (redrawn from [169]). **F**. Danielli’s summary of different transporter models in 1954 (redrawn from [134]).

The idea that cell membranes hosted important metabolism-related carriers or transporters was not totally new (pink boxes in Figure 3). Already in the 1930s, ions had been suggested to cross the membranes thanks to the interaction with some membrane components (Figure 11 F, [158] Ion transport had also tentatively been related to metabolism and respiration [159-161]. In 1947, one early expression of this idea suggested that the membrane fixed the ions to the cell, where the internal respiration was responsible for exchanging the external ions with some other internal ions linked to the cell colloid through the so-called “ion tracks” [128]; it is worth noting that this hypothesis still favored the continuous nature of the cell colloid instead of the diffusion in the interior of the cell (Figure 5). Another transport suggestion was that the membrane had components to which ions could be linked and which could change their conformation to allow the ions crossing the membrane [162]. It was soon suggested that proteins may be the agents of this ion transport [163], although this option was not immediately accepted [164].

Still, in the early 1950s, very little was known about the active transporters themselves. Important progress came from studies on excitable membranes and the transport of non-electrolytes.

#### Excitable membranes

Bernstein’s membrane theory explained action potentials by assuming that transient membrane breakdowns would be responsible for an increased permeability and the subsequent abolishment of the ion gradient ([81,170], purple boxes in Figures 2 and 3). For some decades, the membrane breakdown was a popular mechanism to account for ion crossing even among those authors who thought that membranes had pores [139,142]. Of course, according to this hypothesis, the action potential could not possibly surpass the resting potential. Yet, when direct measures between an internal and an external electrode became possible in 1939, it appeared that the action potential was larger than the resting potential [171]: A mechanism different from the membrane breakdown was necessary to explain these observations. In 1952, after unsuccessful attempts to explain the overshoot based only on K^+^ permeability and membrane changes, Hodgkin and Huxley published in a series of papers that established the sequence of Na^+^ inflow and K^+^ outflow responsible for depolarization and hyperpolarization in axon membranes [172,173].

In his lecture for the Nobel prize, Hodgkin highlighted the fact that, in spite of their radical discovery, little was still known about how these ions flows took place. Since pores were already assumed to exist for the downhill movement of K^+^ across the membrane, this issue drew relatively little attention. On the contrary, since hydrated Na^+^ is larger than hydrated K^+^, how could a pore specifically select for Na^+^ flow and simultaneously avoid K^+^ movement? It was first suggested that Na^+^ used a specific lipid carrier that could cross the membrane when it was depolarized [174], but this track was ruled out because it was contradicted by the reported kinetics of Na^+^ movement [172]. Another related issue also attracted much attention: How could the ions cross the membrane against their gradient after the action potential to recover the values observed at the resting state? This question had already been asked by Overton 50 years before [136,170] and the proceeding answers had been the same as those trying to explain ion distribution asymmetry (see previous section). For instance, Conway suggested that his mechanism based in the Donnan equilibrium could also explain the resting potentials in the nerve [142], but attention progressively moved to active transport as this hypothesis became dominant [157].

Excitable tissues became one of the favorite models for the study of active transport. On one hand, Na^+^ or Na^+^/K^+^ transport in axons was shown to depend on glucose availability and to be affected by several inhibitors of oxydative phosphorylation [175-177] On the other hand, some preliminary reports pointed out to the existence of an ATPase activity located in the lipid fraction of axons [178,179]. In 1957, Skou found a connection between the ATPase and the ion-dependent activity present in the hydrophobic fraction–membrane–of the nerve (for which he also earned a Nobel prize) [180]. This observation launched an overwhelming interest on ATPases that led to a fast accumulation of data covering ATPases from different species, tissues and functions [181]. By 1965, some ATPase features relevant in understanding membrane structure had become conventional wisdom: (1) the active transporters were located in the cell membrane (Figure 11A); (2) they spanned the membrane and were asymmetric, i.e. they showed different affinities in each membrane side; and (3) as they enzymatically hydrolyzed ATP, they were an intrinsic part of cell metabolism.

As we will see next, this progress in transport understanding was paralleled by simultaneous studies on non-electrolyte uptake. The combination of all these different studies reinforced the notion that membrane proteins were enzymes strongly related to metabolism and cell bioenergetics.

#### Membrane-located metabolism and enzyme-like transporters

How non-electrolytes entered the cell was also a matter of speculation for a long time [182]. Initially, if non-electrolyte molecules had crossed the membranes either through their lipid component or through a putative pore, regular diffusion should have been sufficient in predicting their permeability rates. On the contrary, several puzzling observations started to accumulate in the first quarter of the XX^th^ century. For instance, it was shown that the intestine absorbed some sugars more easily than others, even when stereoisomers were compared [182-185], and that some sugars entered the erythrocytes faster when the external concentrations were lower [182,186]. In 1935, Jacques’ precise kinetic analyses showed that permeation was a saturable process ([187] pink boxes in Figure 3). This result advanced the involvement of transporters in non-electrolyte permeation [164]. In the 1950s, a revitalized interest in transporter kinetics revealed that some molecules acted as transport inhibitors [182,188] and that transporters were regulated by their own substrates [189]. These results bolstered the connection between transporters and enzymes not only in eukaryotes but also in bacteria [63,71,166,189-191].

As a result of the accumulation of kinetic, genetic and energetic data, new transporter hypotheses emerged in the 1950s. The classical view was that the transporter was a molecule present in equivalent amounts on both sides of the membrane and able to simply shuttle the attached molecule from one side to the other [162,191]. Figure 11 summarizes the many new transporter modalities envisioned in the 1950s and 1960s [134,192]: (1) a mechanical small transporter propelled from one side of the membrane to the other [193]; (2) a membrane-spanning carrier able to flip-flop; (3) a division of the membrane into rotating segments; or (4) a channel-like protein in which the substrates could specifically interact with different amino-acids along the pore [166]. An additional, provocative and fruitful hypothesis was added by Mitchell in 1957 [167]. His model (Figure 11C) is striking at first because it clearly assumed the transporter to be a protein embedded in the membrane; such a protein was metabolically-driven, enzyme-like and able to swing its attaching site from one side of the membrane to the other according to its conformational changes.

Although indirect, Mitchell’s contribution to membrane understanding should be stressed. As a former student of Danielli, he actively tried to fill the gap between the community working on cell membranes and those who studied the metabolism [194]. In addition to his suggestion for a transporter, his chemiosmotic hypothesis certainly marked a turning point in the way membranes were envisioned. Here too, it would be inappropriate to trace back a detailed account on bioenergetics from their origin (see [134,195] for details). Suffice it to recall that in 1961 the formulation of the chemiosmotic hypothesis accounted for the inclusion of the respiratory chain within the proton-impermeable membrane. According to this hypothesis, the membrane-located respiratory chain employed the energy liberated from redox reactions to translocate protons across the proton-impermeable membrane, and the resulting proton gradient was then available for use by membrane-embedded ATPases to synthesize ATP [168]. Although this suggestion first encountered a vigorous opposition, it became progressively accepted as supplementary studies refined it and some of its predictions were confirmed [134,194,196]. Of particular interest for us was the demonstration that uncoupling agents of ADP phosphorylation and electron transport did not mediate their effect through a direct enzymatic inhibition but through the increase of proton permeability across lipid bilayers [197,198]. This experiment supported the hypothesis that lipids were accessible in the cell surface while contradicting the dominant paucimolecular model in which phospholipid bilayers were insufficient to keep an ionic gradient.

#### An important input from transport studies

In summary, the 1950s and 1960s were full of discoveries seemingly tangential but actually tightly related to cell membranes. These debates concerned the cell permeability, the formation of gradients or the connection with metabolism. The sound transformation that took place in these fields during those years improved knowledge of many membrane components, especially proteins. The pumps, transporters, respiratory chains and ATPases studied in these lines of research required that membrane proteins had access to both sides of the membrane. Although the existence of transmembrane proteins was far from being totally accepted, these hypotheses certainly impacted contemporary ideas on membrane structure at a time not yet dominated by the fluid mosaic model.

### Towards a new membrane model

#### First insights from electron microscopy

Electron microscopy emerged in the 1930s and the first attempts to apply it to the elucidation of cell structure rapidly followed. However, it was not until the 1950s that sharper resolutions allowed the direct observation of cell membranes [88,199,200]. In addition to corroborating their existence, the visualization of cell membranes was expected to provide a powerful tool to investigate their structure. Paradoxically, instead of making things clearer, electron microscopy images launched 15 years of a passionate debate over cell membrane structure (light green boxes in Figure 3).

Indeed, the interpretation of the pictures obtained was difficult and naturally influenced by former conceptions of membrane structure. For instance, Hillier and Hoffman observed the surface of erythrocytes in 1953 and described their membrane as made up of superficial “plaques” attached to an underlying, internal fibrous material [199]. This hypothesis tried to reconcile both the paucimolecular model and the pore theory: on the one hand, the plaques and fibers were thought to correspond to the protein envelope of the membrane of the paucimolecular model; on the other hand, the variable space left among the plaques was suggested to account for the variability of pore diameter in the mosaic model. The same year, 1953, Frey-Wyssling and Steinmann examined the thylakoid surface of plant chloroplasts with the electron microscope [201]. They saw a granular surface and suggested that thylakoid membranes were made up of globular lipoprotein subunits–a possibility that would be extensively explored in the next decade.

The improvement in electron microscopy resolution and sample preparation also allowed for the observation of membrane cross-sections. These pioneering works raised the question of what visual structure should be assumed as the limit of the cell [8]. This question was very challenging given the biological materials that were often used for these observations: Muscle, nerves and microorganisms displayed complicated external structures that made it difficult to determine which layer corresponded to the cell membrane. Even when extracellular structures had been discarded, the interpretation of the remaining superficial layer of the cell was still not self-evident. Cross-sections of that superficial layer revealed the structure known as the “railroad track”: two dense lines separated by a middle, lighter space. Yet, which one among the whole structure or one of the dense parts should be considered as the quintessential cell membrane? In 1959, Robertson compared a collection of cross-section pictures and observed that the whole railroad track was consistently observed in a variety of cells. He thought that the railroad track fit the paucimolecular model, assuming that the dark parts were the protein layers sandwiching and the lighter, the lipid bilayer. As a result, he considered the whole structure to be the cell membrane. Although his hypothesis was not significantly different from the paucimolecular model, he renamed this model the “unit membrane” in order to stress two points: First, that the three layers observed in the electron microscope cross-section shots were part of the same structure, the cell membrane, regardless of the other cell envelopes that might exist; and second, that this structure was universally shared among all biological membranes [202].

#### The diversity of biological membranes

The controversial formulation of the unit membrane hypothesis announced a relentless confrontation in the 1960s between the predominant model at the time (the paucimolecular hypothesis) and the plethora of other membrane explanations suggested by the increasing amount of contradictory information.

Many authors called into question the idea that one unique membrane model could account for all biological membranes because it seemed contradictory to the large membrane diversity that was being discovered at the time [9,203]. For example, it had been observed that the protein-lipid ratio from different membranes could vary from 1:4 to 5:1, suggesting that the amount of membrane proteins was not always enough to entirely cover the cell surface [9]. In addition, the lack of resolution prevented the observation of the railroad track in some bacteria, thus implying that the three-layer structure was either not always visible or not universal [204]. The wide functional diversity of membranes also seemed to contradict the unit membrane: How could the insulating membrane of myelinized nerves have the same structure as the metabolically active membranes responsible for oxidative phosphorylation and photosynthesis [9]?

For some time, the idea that biological membranes could be made up of specific subunits seemed appealing. These hypotheses were natural extensions of the previous thylakoid electron microscopy images by Frey-Wyssling and Steinmann and were popularized by the observation of similar repetitive structures in the chloroplast [205,206], mitochondria [207,208] or even plasma membranes [209]. These hypotheses generally implied that the subunits could be different from one membrane to another and account for membrane functional diversity [9]. As it was becoming more generally accepted that lipids and proteins mainly interacted through their hydrophobic parts, some authors imagined the subunits as dynamic micellar mixes of proteins and lipids [210,211]. Electron microscopy images from viral capsides also lent credit to the view that membranes could be made up of subunits [212]. From an historical perspective, it is noteworthy that these models emphasized the dominant role of proteins as the major structural components of membranes–a fashionable idea at a time when radical developments were being made in molecular biology [213]. The so-called subunits that were being observed most likely corresponded to the functional proteins that dominated the mitochondrial and chloroplastic membranes, for example, ATPases or photosystems.

Given the criticisms, even advocates of the paucimolecular model were compelled to adopt some modifications. For example, in a 1964 review, Brady and Trams incorporated some characteristics that prefigured the modern formulation of the fluid mosaic model: (1) the proteins also penetrated the membrane and, therefore, the membrane was a lipid-protein mosaic; (2) membranes were not homogeneous but could have membrane segments specific for some permeability functions; (3) the lipid components of the membrane were fluid; and (4) membrane formation was ruled by the thermodynamic search of the lowest energy structure [214].

Thus, in spite of Robertson’s unit membrane, the debate on membrane structure steadily grew in the 1960s. Although the debates mostly crystallized around the unit membrane and the subunit-based hypotheses, the change in perspective that allowed the prevalence of the fluid mosaic model was highly impacted by the gathering of conclusive evidence from new techniques.

#### Evidence for a new membrane hypothesis

##### Artificial lipid bilayers

Most studies on amphipathic molecules so far had been focused on molecular monolayers. The first lipid bilayers were artificially prepared in the 1960s by Mueller and collaborators (red boxes in Figure 3), thus providing a much more suitable model to be compared to the biological membranes [215]. Synthetic membranes showed that lipid bilayers were stable even when proteins were totally absent. This observation did not refute the paucimolecular model, but it was an important contradiction to Danielli’s assumption that naked lipid bilayers were too delicate to act as boundaries [216]. The addition of proteins to artificial lipid membranes gave some insight into the capacity of peptides to confer permeability and excitability in membranes [215,217]. Moreover, when artificial lipid bilayers were examined with the electron microscope, it revealed a railroad track similar to biological membranes [218]; this result was at odds with the idea that dense layers corresponded to the proteins coating the membrane in the paucimolecular model. Finally, a better understanding of the parameters that ruled lipid interaction allowed Bangham and collaborators to prepare the first liposomes (i.e. artificial vesicles bounded by a lipid bilayer [219]) in 1965. In fact, liposomes had been observed as early as 1854 by Virchow [220,221]; they had been studied by Lehmann and Reinitzer at the turn of the century [221,222] and lipid suspensions had been made throughout the century, but previous authors had failed to understand that liposomes enclosed an aqueous phase [221]. Now the liposomes would rapidly become important structures in the study of membrane permeability and allowed a fruitful comparison to the biological membranes.

##### Fracture of frozen membranes

The first electron microscopy results had been criticized because the native structures were suspected to be modified by the chemical fixation of the biological material [9]. This criticism was circumvented by the development of the freeze-etching method by Moor and Mühlethaller in 1963 [223]. In this technique, the sample is frozen for fixation and fractured before it is examined with the electron microscope (light green boxes in Figure 3). Branton and collaborators carried out further analyses both with natural and artificial lipid membranes in the late 1960s. They found that the freezing technique kept the hydrophilic interactions at the surface but canceled the hydrophobic forces in the interior of the membrane, thus allowing the membrane to be broken apart between the two lipid bilayers [224,225]. As the interior of the natural membrane became visible, some protuberances were reported on the internal side of each monolayer that were mirrored by depressions in the opposite monolayer. According to these results, the membranes were understood as lipid bilayers intercalated with “globules” that would soon be assimilated into the membrane proteins [226].

##### Membrane fluidity

One of the earliest questions of membrane studies was if the membranes were better depicted as liquids or solids [65,106,227]. A giant step forward was made in 1970 by Frye and Edidin when they fused two cells (one human, one mouse) together in order to monitor the fate of their membranes ([228] dark green boxes in Figure 3). Each cell carried different surface antigens whose movements could be monitored using a fluorescent antibody. After the cell fusion, the two membrane fluorescences became progressively intermixed, thus suggesting that the membrane components were able to freely diffuse in the membrane plane.

##### Protein-lipid interactions

As previously explained, the first definitions of amphipathy, hydrophobicity and hydrophily were sought in the 1910s (dark green boxes in Figure 2 and 3). However, it was not until the 1960s that works like those from Haydon and Taylor emphasized the dramatic role of thermodynamics to determine the structure of biological membranes [229]. Even once the thermodynamic argument had entered the discussion of biological membranes, the first attempts to account for these constraints failed to fully assess their intensity. Hydrophobic and hydrophilic interactions were shown to rule the contact between lipids and proteins [230], yet the early hypotheses often assumed that micellar mixes of lipids and proteins would have been the more stable structures [231], thus favoring the subunit-based models.

A significant breakthrough was achieved when the study of membrane protein conformation became possible. The early paucimolecular model had depicted the coating proteins as globular [115], but in the 1960s the evolution of the model had led to the assumption that the proteins were unwrapped in a conformation similar to a beta sheet [108]. In 1966, several studies showed that the membrane proteins had an alpha or globular conformation rather than a beta structure [232-234]. The alpha helices were suggested to cross the membrane, thus providing a structural framework to the transmembrane proteins that had been predicted in the permeability and transport studies. These works also acknowledged the importance of the thermodynamic constraints to determine the lipid-protein interactions and were especially influential because some of their authors, especially Singer, took part in the formulation of the current version of the fluid mosaic model.

#### Birth and life of the fluid mosaic

In 1971 and 1972, Singer and Nicolson presented their fluid mosaic model of cell membrane structure. The basics of the model have remained the same ever since: the membrane is a lipid bilayer with hydrophilic parts on the sides and hydrophobic parts in the interior; proteins can interact with the surface through transient polar contacts, but a lot of proteins are partially or totally embedded in the lipid bilayer where their hydrophobic parts also interact with the hydrophobic parts of lipids (Figure 1).

In the light of the historical account on cell membrane discovery that I have reported, it is apparent that the success of the fluid mosaic model lay not so much in its originality as in its timeliness and scope: It accommodated most of the evidence available at its time and made predictions that would be demonstrated later.

On the one hand, the model was supported by evidence from different origins: (1) the permeability and transport studies that predicted enzyme-like transmembrane proteins [167]; (2) the apparent lack of lipids to make up complete bilayers [94], thus pointing out to the participation of proteins in the membrane plane; (3) electron microscopy pictures, including freeze-etching studies that suggested the presence of proteins within the membranes [224]; (4) the stability of artificial lipid bilayers that supported them as suitable and sufficient components to make up structures similar in the biological membranes [219]; and (5) the favorable conformations predicted for the membrane proteins [233].

On the other hand, the model was even more influential owing to the assumptions that it highlighted or newly predicted. First, as it was soundly established on thermodynamic grounds, the model enhanced the study of hydrophobic forces, which would subsequently become one of the major explanatory parameters to describe the biological macromolecules [2]. It is important to point out that, more than any generalization from biological observations–as was the case in the unit membrane, for example–the fact that the model is based in universal physico-chemical constraints is the most convincing argument for its general application in biology. Moreover, the acknowledgement of the thermodynamic hydrophobic constraints improved our understanding of membrane proteins, which in turn significantly improved our picture of membranes [6]. Some dramatic landmarks in membrane protein depiction were the early resolution of the first tridimensional structure of a transmembrane protein (the archaeal bacteriorhodopsin, [235]); the development of the patch-clamp technique, which allowed the understanding of single ion channels [236,237]; the discovery of the rotatory catalysis that allows the ATP synthesis by ATPases [238]; and the late discovery of the aquaporins [239,240], which are water channels essential to understanding the water movements that have intrigued cell biologists for more than a century [241]. Interestingly, now that our knowledge on membrane proteins has developed, the importance of lipid interaction for protein folding is becoming clearer and is still a promising line of research for the future [242,243].

Second, since this model is intrinsically fluid, it predicted that the distribution of most molecules in the lateral range would be essentially random, but it also suggested that specific clusters (i.e. microdomains) may form [1,244]. Although mainly ignored for some time, these microdomains have been the subject of intensive research in the last 20 years and the recent introduction of new techniques should continue to improve our understanding of interactions among membrane components [245,246]. Another proof that the fluid nature of membranes remains a subject to be explored further has been the recent interest in state transitions in membranes: In the last decade some biophysicists have studied how membrane transitions from fluid to gel under compression can allow revisiting Hodgkin and Huxley’s mechanism of pulse propagation in excitable membranes [247].

Finally, the asymmetry of membranes has also proven to be a fruitful characteristic to explore. The idea that membranes had different components in the inner and outer sides of the membrane was not new, but this hypothesis was taken one step further because the model provided an explanation: The high, free energy of activation necessary for the hydrophilic part of a membrane component to cross the hydrophobic membrane core prevented the random tumbling [1]. Hence, the asymmetry which was already suspected for the oligosaccharides [248,249] was rapidly extended in the 1970s to lipids, transmembrane proteins or peripheral proteins–for instance those related to the cytoskeleton [235,250-253].

In summary, since its formulation in the 1970s, the fluid mosaic model has been modernized to account for further observations, but it has barely been altered. It remains the most explanatory hypothesis to understand biological membranes.

## Conclusion

### A revision of the cell membrane historiography

Although the subject of cell membrane discovery could certainly be developed further, the information reviewed here should already be enough to point out the major limitations of the majority of previous, short historic accounts on this topic [5-7,9,10,202]. I will try to base my critical discussion on some arguments that Kuhn used to criticize what he called the “sources of authority”. These criticisms are not necessarily related to his well-known concept of scientific revolutions, but I will also briefly tackle the question in order to broaden the perspective of this review.

Kuhn’s concept of scientific revolutions (or paradigm shifts) postulates that science does not only evolve through the gradual accumulation of evidence, as was previously thought, but also through dramatic revisions of previous data that radically change the overall opinion of a given scientific community on a subject [254]. Since its formulation, the paradigm concept has been extensively misused [255] and Kuhn’s ideas have been frequently decried in biology owing to the difficulty in identifying examples of scientific revolutions from specific historical accounts [256]–including the field of membrane transport [134,135]. Interestingly, the formulation of the modern fluid mosaic model has recently attracted some epistemological interest [213]. This recent work suggested that the fluid mosaic model did not drive out its predecessor through a complete revision of available data in a Kuhnian sense; instead, it was the result of a synthesis effort between new pieces of evidence and different models. My historical account supports this synthetic interpretation, as it shows that several mosaic models predated Singer and Nicolson’s hypothesis; the final acceptance of the fluid mosaic did not result from a novel change of perspective, but from the accumulation of supportive data from diverse experiments. Of course, this does not deny the radical contribution of Singer and Nicolson’s model to current biology.

According to this analysis, I think that if the history of the discovery of cell membranes can illustrate some dramatic change in perspective in biology, it should be the transition from the understanding of the cell as a colloid to the bounded, highly concentrated solution currently in use. I think that this transition could be understood in Bachelardian terms as a discontinuity between the pre-scientific era of biology and modern science [257]. Yet, it is not in the scope of this review to carry out a detailed epistemological analysis on the history of the Cell, so I will now move on to the other Kuhnian arguments that I think to be directly relevant to the critical analysis of the cell membrane historiography.

When Kuhn tried to explain the difficulty in accounting for scientific revolutions, he made a particular case for the analysis of the sources of authority, i.e. science textbooks, philosophical works and some outreach presentations of science [254]. He criticized that most of these sources did not provide a comprehensive historical account of the actual events in the way they were understood at the time of their discovery. These texts instead presented individual experiments or thoughts that could be easily viewed as explanatory contributions to the current paradigm. As the aim of those texts was not to provide a detailed account of historical events, they tended to stress some scattered “great heroes of an earlier age” in their relationship to the modern paradigm and to omit contemporary interpretations and opposing ideas to preserve clarity. Although these devices may be adequate for pedagogical purposes, such a presentation distorts the actual historical reconstruction. The stake in this strategy is not only that it may contribute to hide a scientific revolution, as Kuhn feared, but also to make an inaccurate and oversimplified historiography become repeated and established.

If we return to the history of membrane discovery, it is alarming to see how well Kuhn’s criticisms of the “sources of authority” match some insufficiencies in the short historical accounts [5-7,9,10,202]. Some examples of this are: (1) the fact that membrane discovery is systematically presented as a very linear process which obviates the many simultaneous lines of research and debates that have existed throughout history–for example the omission of the coexistence between the paucimolecular and the mosaic models in the first half of the XX^th^ century; (2) the presentation of only a few flagship experiments that supposedly settled the current paradigm, whereas a less hasty analysis shows that the scope of these experiments was repeatedly revisited according to the discovery of new facts, such as different perspectives on osmosis and Overton’s experiments; and, most startling, (3) the incomplete (and misleading) report of some experiments to fit in our current understanding, as in the Gorter and Grendel’s case. These inaccuracies have probably little impact on our current membrane research, but we should be aware of their existence in order to avoid perpetuating a historically questionable timeline.

I hope that this review will encourage other scholars to critically revise our short accounts on fields traditionally underrepresented in the History of Science studies as, for example, cell biology, microbiology and biochemistry.

### Perspectives from a historical background

The historical analysis of the formulation of the current membrane model is not only relevant to those interested in membranes: it may also provide some lines of reflection on early evolution, the minimal cell concept, the origins of life and synthetic life. All these subjects question our vision of the cell, and the membrane is arguably one of the most essential components of the unit of life concept.

To begin with, we can step back to ask the question of the very necessity of the unit of life notion. It has been argued that the Cell Theory stands on the biological atomism, which postulated the existence of a basic indivisible unit of life well before any precise description of this unit could be made [258]. According to this appealing analysis, the atomistic idea remained implicit along with the new discoveries that led to and established the Cell Theory. Even the past and present opponents to the Cell Theory seem to agree with the atomistic idea, as their arguments challenge the relevance of the Cell as the unit of life, but not the existence of a “unit” itself [258]. Determining the appropriateness of biological atomism is a deep epistemological question, which is not the matter here. Yet, if we accept that the unit of life is a reality that we can study in spite of the diversity of opinions on the identity of this unit, the current preferred candidate for this position among biologists remains the Cell. Therefore, as all known cells are bounded by cell membranes, understanding the importance of these structures becomes crucial to our current definition of the unit of life.

The historical analysis supports the progressive acknowledgement in the last decades of the importance of cell boundaries in the fields of early evolution and the origins of life [259-261]:

1. As the unit of life, the cell entails some kind of identity that differentiates it from other cells and from the environment. Since it also has a composite structure, the cell requires a mechanism to keep all its components together. Historically, two mechanisms have been envisioned: either all the components remain together because they establish direct interactions in a physical network (the colloid chemistry) or they are compartmentalized by some structure. It is important to remember that even after the discovery of the cell membrane, the colloid hypothesis survived many years and was only replaced when the biological macromolecules started to be analyzed as discrete structures. This is relevant to the origins of life, as well as synthetic life studies, because it supports current thinking that the compartmentalization is one of the very basic characteristics of any cell [259-261], no matter how primitive or minimal it may be.

2. The membrane embodies one of the main paradoxical characteristics of life: a cell is a system dependent on external compounds and energy to keep the differences that it maintains with the same environment where it gets its raw material. Although membranes were thought for a long time to be passive structures that just allowed solutes to diffuse across them, we now know that modern membranes are necessarily endowed with the ability to control the entry and exit of molecules depending on their needs, even sometimes against the chemical gradients. According to this observation, it seems important to include a thought about (active) transport mechanisms in all works trying to describe the nascent life. This does not mean that complicated structures, like proteins, had to be present from the very start of compartmentalization. For instance, transmembrane gradient formation based on membrane dynamics and alternative transporter molecules (e.g. RNA molecules) have been studied in recent years [262-265]. We can expect that the awareness of the importance of (active) transport for all cells will soon attract more attention to this fascinating subject from the researchers working on the origins of life.

3. Contrary to early assumptions about membranes, one of the major foundations upon which the fluid mosaic model is built is their ever-changing dynamic structure. This allows modern membranes to constantly change their activities according to the requirements of the cell and it is likely that the same could have occurred in early membranes. Such a hint is promising because it could intersect with the increasing interest of the origins of life field in studying the changing abilities of membranes made up from mixed amphiphile solutions [266-268].

4. Finally, there are at least two fundamental aspects of membranes that have not been discussed in this review because their contribution to the understanding of membranes was low, but they cannot be neglected when referring to membrane contributions to cells. These are the division of membranes and their role as transducers of messages from the environment. Although membrane division has already attracted some attention in the context of the origins of life [269], very little is known about the interactions among early cells. Hopefully, both subjects will be further explored in the near future.

## Revierwers’ comments

I thank the reviewers for their comments. The manuscript has been revised twice taking into account their remarks.

### First round

#### Referee 1, Dr. Étienne Joly

This manuscript by Jonathan Lombard provides a very thorough and detailed historical account of the evolution of the notion of cell boundaries over the 300 years that spanned between the initial recognition that living organisms were comprised of cells, in the middle of the 17th century, and 1972, with the advent of the fluid mosaic model, and the now generally accepted view that all living cells are surrounded by biological membranes made of lipid bilayers. Although I am not competent to judge the accuracy of this historical report, and would not know if equivalent works have been published previously, I feel that this manuscript should represent a valuable addition to the field, and that the final parts of the manuscript, and the discussion in particular, raise several interesting questions and prospects.

All these good things being said, despite the tremendous amount of historical work which has clearly gone into assembling this manuscript, I must admit that I have found the reading of this manuscript to be rather cumbersome, and even very hard work for the early historical parts. I have communicated numerous corrections and editorial suggestions to the author directly, and hope that this will help him to produce a revised manuscript that will be easier to read, and thus more useful for the scientific community.

*Author’s response: I thank the referee for his constructive suggestions to make the review easier to read. The overall structure of the new version of the manuscript has remained unchanged, but I have rewritten many paragraphs and shuffled some sections in order to clarify their message. I have also tried to make the transitions between paragraphs more fluent and I have removed redundant information to make more obvious the common thread of the text. The new version of the paper has been checked by a professional journalist native in English who helped me to make the reading smoother. Thus, I think that the current version of the manuscript should be more easily readable than the first version.*

#### Referee 2, Eugene V. Koonin

In this long paper, the author describes, in considerable detail, the history of biological membrane research, with an emphasis on the role of the membrane as the active cell boundary that determines what gets into or out of the cell and what remains inside or outside. It is rather surprising to read, as a submission to a biology journal, an article that earnestly addresses the intricacies of the history of research in a particular field, without making much effort to formulate any new concept on the functions or evolution of membranes. This is not a criticism, the history of concepts and misconceptions is useful and interesting in itself.

What is missing, from my perspective, in this article, is any discussion of organellar and other intracellular membranes as well as membranes found in virions. Even if the main emphasis is on cell boundary, contrasting the features of plasma membranes to those of these distinct membranes, especially in the context of the “active membrane” concept, could be useful.

*Author’s response: I thank the reviewer for this comment. The intracellular membranes are indeed a fascinating subject of study and I would have been glad to introduce them in my review. Nevertheless, I think that this manuscript is already very long and, as the first referee has noted, the main challenge here is to remain pertinent in order to keep the readers’ attention. Therefore, I have preferred to stick to the core of the subject, namely the origins of the membrane concept and the fluid mosaic model. As for other fields, I referred to intracellular membranes only when their study directly contributed to the storyline that I was trying to highlight in this paper. But I will consider the possibility of preparing a separate review about the history of intracellular membranes.*

To me, the following point: “As the membrane is the cell element which decides what may or may not enter the cell, these entities require active membranes. From my point of view, this active membrane vision contradicts some hypotheses on the origins of life that favor a progressive increase in membrane tightness [264,265]” looks like a non sequitur. I do not see how these hypotheses clash with the “active membrane” concept and furthermore, have a difficulty imagining the origin of protocells without gradual tightening of the membrane.

*Author’s response: This sentence has been totally removed from the new version of the manuscript. In order to account for the suggestions made by the third reviewer, the final section of the conclusion has been considerably changed.*

#### Referee 3, Armen Mulkidjanian

The review by Lombard is a nice survey on the evolution in understanding the nature of cell envelopes in the course of past three centuries. It is an entertaining reading indeed and there is not much to comment.

Still several points deserve mentioning:

1. **Introduction, line 31ff**: The reader can get an impression that any lipid molecules tend to join into a membrane bilayer as the most thermodynamically stable structure. This is not the case. One of conditions of forming a bilayer is a match between the sizes of the hydrophobic and hydrophilic parts of the molecules involved [1]. A bilayer is formed when these parts are of approximately the same “width”. If the hydrophilic head is larger, then the most stable structure is not a bilayer, but a micelle. If, on the contrary, the hydrophobic part is “broader”, then a reverse micelle or a so-called hexagonal phase are the thermodynamically most stable structures.
  *Author’s response: The text has been changed to account for this comment. Now it says: “When they are diluted in water, amphiphiles spontaneously adopt the most thermodynamically stable molecular structure, namely the one that maximizes both hydrophilic and hydrophobic interactions [2]. These interactions may be affected by several parameters, such as the chemical nature of the molecules, their size, the salinity and pH of the solution. In biological conditions, cell phospholipids form a bilayer in which hydrophobic tails face each other in the core of the structure whereas the hydrophilic heads interact with the water molecules in the sides (Figure*1*).”*
2. **Section*“Osmotic studies and artificial membranes”,*line 290 ff**. The story of “precipitation membranes” is quite exciting. It might be interesting to know whether they remained a historic curiosity or there is a historic connection with modern chemical engineering of nanoscale systems.
  *Author’s response: I thank the referee for this comment. I had never thought about precipitation membranes in the perspective of nanostructures. Unfortunately, I do not know anything about chemical engineering of nanoscale systems, and this would be a subject too distant from the rest of the review to be included in it. But I appreciate the comment and I will keep it in mind for the future.*
3. **Sections** “***Membrane-located metabolism and enzyme-like transporters”*and “*An important input from transport studies”***: It should be noted that some results directly related to the membrane structure, apparently, were not recognized by the community right away because their authors were interested in quite different subjects and therefore did not put emphasis on these results. While Singer, as it can be followed from his publications, was particularly interested in understanding the nature of biological membranes, Peter Mitchell was more interested in the processes of membrane transport and mechanisms of energy conversion. Thereby Mitchell - and his colleagues - needed (implicitly) some working model of a biological membrane. The Figure from the Mitchell’s paper of 1957 [2], which is kindly redrawn by the author as Figure 10C, provides a quite modern presentation of a biological membrane with integral membrane proteins embedded, without any protein layers that flank the lipid bilayer, not to mention that the hypothetical mechanism for a membrane permease, as shown in the figure, has been later shown to be valid for the vast majority of membrane permeases indeed. Thereby Mitchell did not make any statements on the nature of biological membranes. On the other hand, Singer, in his PNAS paper published nine (!) years later [3], still confronted the scheme with protein layers covering the lipid bilayer and provided evidence on existence of alpha-helical protein that cross the bilayer. Furthermore, the very fact that chemically quite different, small proton-carrying molecules could uncouple oxidative phosphorylation by diffusing across membrane bilayer (as shown first by Skulachev and co-workers [4], this reference should be included), implies the presence of free lipid patches accessible from the water phase for these small molecules; it is across such patches that the uncoupling molecules could diffuse. Again, Skulachev and co-workers did not discuss the presence of these free patches because they were interested in understanding the mechanism of energy conversion. Still, their working model of the membrane should have been that of a mosaic membrane. Hence, the bioenergeticists, particularly the “Mitchellian” community, took the mosaic nature of the membrane and the existence of asymmetrically organized integral membrane proteins as granted; this happened several years before Singer and Nichols published their seminal paper. However, the studies of energy conversion did not provide - at least at that time - any information on the lateral motility of proteins in the membrane. It is not incidental that the paper of Singer and Nicolson [5], in addition to an extended analysis of literature data, also provided experimental evidence of the protein mobility in native membranes. This was the truly new piece of evidence that helped to compile a whole picture of a fluid, mosaic membrane.
  Because of this reasoning, I would suggest to move the sections “***Membrane-located metabolism and enzyme-like transporters”*and “*An important input from transport studies”*** to the next chapter (“Towards a new membrane model”).
  *Author’s response: As the referee says, Mitchell and other authors interested in bioenergetics did not directly address the issue of membrane structure. Their work certainly influenced the way membranes were considered, but it was not used as a piece of evidence to directly oppose the predominant paucimolecular model. That is the reason why I have included Mitchell and others’ work in the chapter “Insights from the movements across the membrane” together with other researches on transport that also indirectly influenced the perspective on membranes. I think that moving this section to the next chapter (“Towards a new membrane model”) would be difficult to fit in my account, so I have preferred to keep it as it originally was. The main modification that I have made in the “Membrane-located metabolism” section has been reducing it in order to make the reading more fluent, according to the advice of Referee 1. The citation to Skulachev and colleagues has been included in the new version of the manuscript.*
4. **Section “A revision of the cell membrane historiography”**. I am not quite happy with invoking the Kuhn’s concept of scientific revolution in relation to the historic narrative on the evolution of membranes. Thomas Kuhn has developed his theory based on the history of physics, therefore his model does not work as nicely with less exact subjects. There is an extensive sociological study on the “scientific revolution” in bioenergetics [6]; the book exemplifies that the paradigm change happened in a rather chaotic, “non-Kuhnian” manner. I suspect that the same can be said about the revolution in “membranology”. As argued above, many scientists, particularly the first partisans in the field of membrane bioenergetics, have used an almost correct “working model” of a membrane already a decade before it was formally presented by Singer and Nicolson. This kind of development can be hardly imagined in physics. I would suggest skipping or modifying this section.
  *Author’s response: I agree with the referee in the fact that the acceptance of the fluid mosaic model should not be considered as a scientific revolution in Kuhn’s terms. Apparently the message was not clear enough in the first version of the paper, so I have modified the text to emphasize more this point. I would like to stress that my main objective in mentioning Kuhn’s work was using his criticisms about the “sources of authority” in science, not saying that the historical process described here was a Kuhnian revolution.*
5. **Section “Perspectives from a historical background”:** Here the author writes that “*one of the dramatic historical changes in thought about the cell membranes was the acknowledgement that biological membranes were not inert but very dynamic structures*.” Subsequently, it is stated that “*As the membrane is the cell element which decides what may or may not enter the cell, these entities require active membranes. From my point of view, this active membrane vision contradicts some hypotheses on the origins of life that favor a progressive increase in membrane tightness* [264,265]”. Finally, it is pointed out that “*historical analysis reminds us that totally passive membranes do not seem very suitable to explain the active processes characteristic of living cells, apart from the transport of gases and the tiniest molecules. This does not imply that early membranes had to have a developed protein apparatus for selective transport, but whatever the nature of the specific transporters present in the primordial cells, their functions are arguably very ancient. I wonder if the presence of a dynamic membrane able to actively transport molecules and maybe metabolize them should not be considered as one of the requirements for a primordial entity to be considered as alive”*.

From my opinion, several different subjects are mixed up in this passage. They deserve being sorted out.

- The very fact that proteins and lipids diffuse within the liquid matrix of the bilayer means that biological membranes are *dynamic*. As far as I know, the dynamic nature of biological membrane has not been challenged neither by authors of the mentioned references 262 and 263 [7, 8]. nor by anyone else in recent years.
- According to the author, the membrane is *active* because “membrane is the cell element which decides what may or may not enter the cell”. Here, it is easy to envision a solid state membrane with pores of particular size that will “decide” what may or may not pass. This membrane, although *non-dynamic*, could be considered as *active* using the author’s own definition.
- The apparent usage of *dynamic* and *active* as interchangeable terms by the author makes this passage even more confusing.
- The models in the mentioned references 262 and 263 [7, 8], which are accused in implying passive membranes, in fact, build on an assumption that the very first membranes could be impermeable to large polymeric molecules but leaky to small molecules. Accordingly, these membranes could already decide “what may or may not enter the cell” and should be categorized as *active* according to Lombard. This vision of primordial membranes is not quite original and could be traced to the studies of Deamer, Luisi, Szostak, Ourisson, Nakatani and their co-workers [9-17]. These authors argued that abiotically formed amphiphilic molecules, most likely, fatty acids of phosphorylated, branched polyprenol-like compounds [10, 12, 14, 18, 19], which may have enveloped the first cells, could not be as sophisticated as modern two-tail lipids. Vesicles formed from such molecules are million times more leaky that vesicles from modern, two-tail lipids [18]. Hence, such vesicles could trap large polymers but not small molecules and ions. This leakiness, however, could have been a key advantage. In the absence of membrane-embedded transport proteins, which apparently could emerge only on a relatively late step of evolution, after the emergence of water soluble proteins [20], the membrane leakiness should have enabled the “consumption” of diverse small molecules by the first cells. In turn, this would favor the development of systems that could trap small molecules by attaching them to intracellular polymers - and thus preventing their escape. Hence, leaky membranes could have driven the emergence of different polymerases, including the translation system. The feasibility of such a mechanism has been experimentally shown [13]. Accordingly, even the first abiotically formed membranes mentioned in [7, 8] should have been both *dynamic* and *active* (according to Lombard).

It seems that the author, besides confusing the terms “active membrane” and “dynamic membrane”, falls victim to the popular, albeit erroneous, notion that an ionic disequilibrium across the cell membrane is an essential requirement for life. The leakiness to ions *per se* neither makes a cell membrane *passive* nor obligatory kills the cell. Even a leaky membrane will faithfully maintain all disequilibria that concern large polymeric molecules. Modern cells are quite robust concerning the tightness of their membranes. The well-known study by Harold and Van Brunt of 1977 has shown that collapsing of the transmembrane electrochemical potential in bacteria by adding diverse ionophores did not affect the growth rate of these bacteria as long as they were in a rich, K^+^-containing medium [21]. The same treatment, however, blocked the growth of the bacteria in a rich, but Na^+^-containing medium. These data show that the high tightness of modern cell membranes is crucial only for one particular function, namely keeping the K^+^/Na^+^ ratio within the cell above one and providing energy for the respective ion pumps. The prevalence of K^+^ over Na^+^ within the cell is crucial indeed for protein synthesis and some other cell functions [8, 22]. When prevalence of K^+^ over Na^+^ is taken care of, bacterial cells can grow even with “bad”, leaky membranes.

I would suggest a truncation or a major modification of the section “**Perspectives from a historical background”.**

*Author’s response: I thank the referee for pointing out the distinction between “dynamic” and “active” membranes. In a previous version of this manuscript, I used both terms indistinctly to refer to what should only be considered as active transport. I have corrected this error and, according to the comments of this reviewer, I have shuffled the section “perspectives from a historical background”. I have removed all mentions to the hypothesis of the progressive “tightening” of membranes and tried to only discuss points that could easily be related to recent experiments in the field of the origins of life.*

### Second round

#### Referee 1, Dr. Étienne Joly

This manuscript by Jonathan Lombard provides a very thorough and detailed historical account of the evolution of the notion of cell boundaries over the 300 years that spanned between the initial recognition that living organisms were comprised of cells, in the middle of the 17th century, and 1972, with the advent of the fluid mosaic model, and the now generally accepted view that all living cells are surrounded by biological membranes made of lipid bilayers. Although I am not competent to judge the accuracy of this historical report, and would not know if equivalent works have been published previously, I feel that this manuscript should represent a valuable addition to the field, and that the final parts of the manuscript, and the discussion in particular, raise several interesting questions and prospects.

Although I had found the reading of the initial version of this manuscript to be rather cumbersome, I must say that I am impressed by how improved and easier to read the revised version has become, and I am now confident that it represents a very useful addition to the scientific literature.

*Author’s response: I thank the referee for his previous comments, which were very useful to prepare the second version of the manuscript.*

#### Referee 2, Eugene V. Koonin

I have no further comment and believe that the paper is ready for publication.

*Author’s response: I thank the referee for his appreciation and previous comments.*

#### Referee 3, Armen Mulkidjanian

I appreciate the author’s efforts on improving the paper in response to the suggestions of the reviewers. Specifically, in response to my comment #5, the author has dramatically truncated and rewritten the section “Perspectives from a historical background”. Still, the revised version of the manuscript contains the following paragraph, which in my opinion is bound to mislead the reader: “Although membranes were thought for a long time to be passive structures that just allowed solutes to diffuse across them, we now know that modern membranes are necessarily endowed with the ability to actively transport molecules according to their needs. According to this observation, it seems important to include a thought about active transport mechanisms in all works trying to describe the nascent life. This does not mean that complicated structures, like proteins, had to be present from the very start of compartmentalization. For instance, recent works have shown that transmembrane gradients across lipid bilayers could spontaneously arise in prebiotic conditions [262,263]. It has even been argued that RNA molecules could have acted as transporters [264,265]. We can expect that the growing awareness of the importance of active transport in early life will soon attract more attention for this fascinating subject”.

From this wording, the reader can get an impression that refs. 260–263 (in the revised manuscript) contain experimental evidence of a spontaneous formation of transmembrane gradients across lipid bilayers in prebiotic conditions. However, this is not the case. The word “gradient” - in the given context - implies a transmembrane difference in concentrations. Such a difference could not arise spontaneously, i.e. without any free energy input (the second law of thermodynamics). Accordingly refs. 260–263 do not provide any experimental evidence for spontaneous emergence of transmembrane gradients. Refs. 261 [23] and 262 [24] contain some interesting speculations, but no experimental data. The ref. 263 [25] contains experimental evidence that a particular RNA aptamer could facilitate the passive translocation of tryptophan molecules across a lipid bilayer; it is important to note that upon such a translocation a concentration gradient can only decay. And, finally, the ref. 260 by Chen and Szostak [26] describes the formation of proton gradient across fatty acid vesicles upon addition of “new” fatty acid molecules. However, Chen and Szostak themselves emphasized that the formation of the proton gradient was driven by thermodynamically favorable incorporation of the new hydrophobic fatty acid molecules into the membranes of the vesicles. Hence, the formation of the proton gradient was not spontaneous. Chen and Szostak observed the formation of the transmembrane pH difference under very special conditions where polar, charged, and bulky arginine molecules were used as the only cations in the medium. Otherwise the pH difference could not arise because the fatty acid membranes were leaky both to protons and to such cations as K+ or Na+. Conditions where arginine molecules are the only positively charged ions in the medium are not “prebiotic” conditions because they cannot be related to any imaginable geological settings. The seminal article of Chen and Szostak, in fact, shows that:

1. a bilayer made of primitive, single-chain lipids is leaky both to protons and to monovalent cations.
2. such a bilayer represents a barrier only for large polar molecules carrying several charges.

To summarize, the author of the review fails to provide any experimental evidence of a spontaneous formation of transmembrane gradients across primitive lipid bilayers in prebiotic conditions. Because of the ability of small ions, such as K+, Na+, Cl-, to diffuse freely in and out of nascent cells, active transport of such ions, if any, should have remained futile until the emergence of two-tail phospholipids and ion-tight bilayers, which seemingly happened independently in bacteria and archea [27]. Active transport of small ions in early life may indeed be a “fascinating subject” but so far it remains only an author’s speculation.

##### Author’s response

*I thank the reviewer for his careful lecture of the article. I did not mean this part of the paper to be as controversial as the reviewer seems to find it to be. The main objective of my review is to provide a new survey on the history of the discovery of cell membranes. I think that this subject is even more exciting when it is placed in the context of modern debates about early membranes. But the issue of early membranes is a wide and hot topic and this review is not the place to discuss it into detail. This review was never intended to provide experimental data about transmembrane gradient formation in early membranes.*

*Although I don’t agree with all the arguments of the reviewer (especially, I don’t understand why there should be any contradiction for a reaction between being thermodynamically driven and spontaneous), I don’t think this article is the place to go into the specifics of the hypotheses about the early evolution of membranes. As a result, I have reworded the problematic paragraph as follows: “Although membranes were thought for a long time to be passive structures that just allowed solutes to diffuse across them, we now know that modern membranes are necessarily endowed with the ability to control the entry and exit of molecules depending on their needs, even sometimes against the chemical gradients. According to this observation, it seems important to include a thought about (active) transport mechanisms in all works trying to describe the nascent life. This does not mean that complicated structures, like proteins, had to be present from the very start of compartmentalization. For instance, transmembrane gradient formation based on membrane dynamics and alternative transporter molecules (e.g. RNA molecules) have been studied in recent years* [262-265]*. We can expect that the awareness of the importance of (active) transport for all cells will soon attract more attention to this fascinating subject from the researchers working on the origins of life.”. I hope that the reviewer will find these changes satisfactory.*

##### Reviewer’s request

I request explicitly that my comments to the manuscript should be published together with the complete reference list [that I provided].

*The following are the references cited by the reviewer in his comments:*

1. Israelachvili JN, Marcelja S, Horn RG: Physical principles of membrane organization. Q Rev Biophys 1980, 13(2):121–200.

2. Mitchell P: A general theory of membrane transport from studies of bacteria. Nature 1957, 180(4577):134–136.

3. Lenard J, Singer SJ: Protein conformation in cell membrane preparations as studied by optical rotatory dispersion and circular dichroism. Proc Natl Acad Sci U S A 1966, 56(6):1828–1835.

4. Skulachev VP, Sharaf AA, Liberman EA: Proton conductors in the respiratory chain and artificial membranes. Nature 1967, 216(5116):718–719.

5. Singer SJ, Nicolson GL: The fluid mosaic model of the structure of cell membranes. Science 1972, 175 (4023):720–731.

6. Gilbert GN, Mulkay M: Opening Pandora’s Box: Cambridge University Press; 1984.

7. Szathmary E: Coevolution of metabolic networks and membranes: the scenario of progressive sequestration. Philos Trans R Soc Lond B Biol Sci 2007, 362(1486):1781–1787.

8. Mulkidjanian AY, Bychkov AY, Dibrova DV, Galperin MY, Koonin EV: Origin of first cells at terrestrial, anoxic geothermal fields. Proc Natl Acad Sci U S A 2012, 109(14):E821-830.

9. Hargreaves WR, Mulvihill SJ, Deamer DW: Synthesis of phospholipids and membranes in prebiotic conditions. Nature 1977, 266(5597):78–80.

10. Deamer DW: The first living systems: a bioenergetic perspective. Microbiol Mol Biol Rev 1997, 61(2):239–261.

11. Deamer DW: Origins of life: How leaky were primitive cells? Nature 2008, 454(7200):37–38.

12. Szostak JW, Bartel DP, Luisi PL: Synthesizing life. Nature 2001, 409(6818):387–390.

13. Mansy SS, Schrum JP, Krishnamurthy M, Tobe S, Treco DA, Szostak JW: Template-directed synthesis of a genetic polymer in a model protocell. Nature 2008, 454(7200):122–125.

14. Nakatani Y, Ribeiro N, Streiff S, Desaubry L, Ourisson G: Search for the most primitive membranes: some remaining problems. Orig Life Evol Biosph 2012, 42(5):497–501.

15. Streiff S, Ribeiro N, Wu Z, Gumienna-Kontecka E, Elhabiri M, Albrecht-Gary AM, Ourisson G, Nakatani Y: “Primitive” membrane from polyprenyl phosphates and polyprenyl alcohols. Chem Biol 2007, 14(3):313–319.

16. Ourisson G, Nakatani Y: The terpenoid theory of the origin of cellular life: the evolution of terpenoids to cholesterol. Chem Biol 1994, 1(1):11–23.

17. Gotoh M, Sugawara A, Akiyoshi K, Matsumoto I, Ourisson G, Nakatani Y: Possible molecular evolution of biomembranes: from single-chain to double-chain lipids. Chem Biodivers 2007, 4(5):837–848.

18. Mansy SS: Membrane transport in primitive cells. Cold Spring Harb Perspect Biol 2010, 2(8):a002188.

19. Dibrova DV, Chudetsky MY, Galperin MY, Koonin EV, Mulkidjanian AY: The role of energy in the emergence of biology from chemistry. Orig Life Evol Biosph 2012, 42(5):459–468.

20. Mulkidjanian AY, Galperin MY, Koonin EV: Co-evolution of primordial membranes and membrane proteins. Trends Biochem Sci 2009, 34(4):206–215.

21. Harold FM, Van Brunt J: Circulation of H+ and K+ across the plasma membrane is not obligatory for bacterial growth. Science 1977, 197(4301):372–373.

22. Mulkidjanian AY, Bychkov AY, Dibrova DV, Galperin MY, Koonin EV: Open questions on the origin of life at anoxic geothermal fields. Orig Life Evol Biosph 2012, 42(5):507–516.

23. Lane N, Martin WF: The origin of membrane bioenergetics. Cell 2012, 151(7):1406–1416.

24. Vlassov A: How was membrane permeability produced in an RNA world?Orig Life Evol Biosph 2005, 35(2):135–149.

25. Janas T, Yarus M: A membrane transporter for tryptophan composed of RNA. RNA 2004, 10(10):1541–1549.

26. Chen IA, Szostak JW: Membrane growth can generate a transmembrane pH gradient in fatty acid vesicles. Proc Natl Acad Sci U S A 2004, 101(21):7965–7970.

27. Koonin EV, Martin W: On the origin of genomes and cells within inorganic compartments. Trends Genet 2005, 21(12):647–654.
